# The role of surface air temperature over the east Asia on the early and late Indian Summer Monsoon Onset over Kerala

**DOI:** 10.1038/s41598-019-47945-4

**Published:** 2019-08-13

**Authors:** Devanil Choudhury, Debashis Nath, Chen Wen

**Affiliations:** 1 Center for Monsoon System Research, Institute of Atmospheric Physics, Chinese Academy of Sciences, 9804, 40-Hua Yan Li, Beijing, 100029 P. R. China; 2 University of Chinese Academy of Sciences, 19A-Yuquan Road, Beijing, 100049 P. R. China

**Keywords:** Atmospheric dynamics, Scientific data

## Abstract

This study investigates the physical mechanism for late Indian Summer Monsoon onset over Kerala (MOK). 14 early and 9 late onset years are selected based on the criteria when the onset is 5 days or more prior and after normal onset date (i.e 1^st^ June according to India Meteorological Department) respectively. Then, we perform composite analyses of mean May monthly and daily evolution during early and late onset years to examine the differences in monsoon circulation features prior to the MOK. We find that advection of Surface Air Temperature (SAT) from the northern to the southern China and the eastern Tibetan Plateau (TP) plays an important role to modulate the MOK processes. In the late onset years, more low-level jet (LLJ) from the Bay of Bengal (BOB) divert towards the east Asia before the onset, which is due to an extension of the low sea level pressure and high SAT over the east Asia (eastern TP, east-central China). This strengthens the low-level convergence and upper level divergence over the eastern TP and southern China. As a result, a significant amount of moisture from the BOB is transported towards the eastern TP and southern China. Thereby, a comparatively weaker LLJ and deficit low-level moisture supply over the eastern BOB maintain the key roles in modulating the MOK processes.

## Introduction

The Indian Summer Monsoon (ISM) onset is one of the most significant and anticipated meteorological phenomenon, which marks the commencement of rainy season in India. During the ISM season (June-July-August-September) India receives about 80% of the total annual precipitation^1^. The ISM onset represents significant transitions in the large-scale atmospheric and oceanic circulations in the Indo-Pacific region, and any kind of uncertainty or drastic shift in the ISM onset can have huge impact on the agriculture and food security in the Indian subcontinent. The monsoon onset is associated with a sudden and consistent increase in the daily rainfall over the Kerala, steady increase in convection over the southeast Arabian Sea (AS) and strong cross-equatorial Low Level Jet^2^ (LLJ). It also increases the moisture convergence over the AS and involves in the development of a low pressure area stretches from the southeast AS to Gujarat coast, known as ‘monsoon onset vortex’^3^. It is well known that the land-sea thermal contrast plays a major role in modulating the ISM onset. Yanai *et al*.^4^ suggested that the Asian Summer Monsoon (ASM) onset is an interaction process between the Plateau-induced circulation and the circulation associated with northward migrating rainband^4^. Li and Yanai (1996) argued that the ASM onset is coincident with the reversal of the meridional temperature gradient in the upper troposphere of the southern Tibetan Plateau (TP). As in the pre-monsoon season (March-May), the upper tropospheric temperature (200–500 hPa) in the TP region increases due to release of sensible heat fluxes from the land surface, and this abrupt warming leads to an increase in meridional temperature gradient in the TP region (as south and north of TP remain relatively cooler during this time)^5^. The sea surface temperature (SST) anomaly over the Indian and Pacific Ocean also influences the ISM onset.

During January to May, since the center of the tropical warm pool region is shifted from the south-west Pacific to the North Indian Ocean (NIO)^6–8^, the warm pool makes favorable conditions for Monsoon Onset over Kerala (MOK) by building up moisture over the NIO and surrounding Pacific Ocean^9,10^. However, Sabeerali *et al*.^11^ demonstrated that warmer Indian Ocean SST is closely linked with delayed monsoon onset^12^. Sabeerali *et al*.^11^ and Pradhan *et al*.^12^ also indicated that warmer SSTs over central Pacific, eastern Pacific and Indian Ocean are highly related to the late monsoon onset by decreasing convection and latent heat release over Indian landmass^11,12^. This is associated with the descending (anti-cyclonic circulation) and ascending branch (cyclonic circulation) of Walker circulation over India and central Pacific, respectively.

The normal ISM onset date, as determined by the India Meteorological Department (IMD) is 1^st^ June, with an interannual variability of 8–9 days. The changes in the onset date have significant impact on the socio-economy of the Indian subcontinent due to variability in the seasonal mean monsoon rainfall^13^. Since 1972, the earliest and the delayed onset had occurred on 11^th^ May of 1918 and 18^th^ June of 1972, respectively. Joseph *et al*.^2^ showed that the delays in MOK are strongly associated with the El Niño. They argued that in the pre-monsoon season, there are warm SST anomalies in the south of equatorial Indian and Pacific Oceans and cold SST anomalies in the north of tropical and subtropical oceans. The difference in SST induces the variability in monsoon onset by affecting the movement of the Inter Tropical Convergence Zone (ITCZ)^3^. Preenu *et al*.^14^ studied the variability of MOK dates from 1870–2014 and the results are consistent with Joseph *et al*.^2^. They discussed that the large scale SST anomalies are similar to the canonical El Niño and Southern Oscillation (ENSO) pattern and are associated with the inter-annual variability of the MOK^3,14^. Zhou and Murtugudde (2014) reported that strong northward-propagating intra-seasonal variability (ISVs) may trigger an early onset by transporting moisture and momentum flux northward, which may trigger more atmospheric instabilities and convection in the tropics^15^. The northward propagation of ISVs depends on the vertical wind shear, and the strength of ISV is enhanced when the shear turns easterly in northern hemisphere^16^. Sahana *et al*.^17^ showed a shift in ISM onset during 1976/1977 and reported a prominent delay in ISM onset date based on the Hydrologic onset and Withdrawal Index (HOWI). They argued that the delays in the onset after a shift in ISM onset during 1976/77 are due to delay in the development of the easterly vertical shears. It reduces the northward propagation of ISVs during May-June, therefore, limiting the moisture supply from the Indian Ocean to the AS^17^. Mao and Wu (2007) indicated that an early (late) Bay of Bengal (BOB) Monsoon Onset is associated with less (more) TP snow accumulation during the preceding winter^18^. During the years of high (low) Eurasian snow amounts in spring/winter followed by deficient (excess) ISM rainfall^19^. Also, it is shown that an excess summer monsoonal rainfall over Asia corresponds to low April snow depth over the Eurasian region^20^. Further, Panda *et al*.^21^ explained that because of the west Eurasian snow depth anomalies, the mid-latitude circulation undergoes significant changes, which in turn lead to weak/strong monsoon circulation during deficient/excess ISM rainfall respectively^21^. Despite several researchers investigate on the ISM and its onset variability, the huge complexities in large-scale evolution of the summer monsoon circulation and its coupling between ocean and atmosphere still demand more robust investigations to address the dynamical meteorological drivers controlling the MOK variability.

In order to find the differences between large-scale circulation characteristics associated with some early and late MOKs and its evolution, an attempt has been made in this study by selecting 14 early and 9 late onset years from 1979 to 2017. To carry out the investigation, some key meteorological variables are systematically analyzed here.

## Results

### Mean composite atmospheric circulation in May

**Figure 1:**
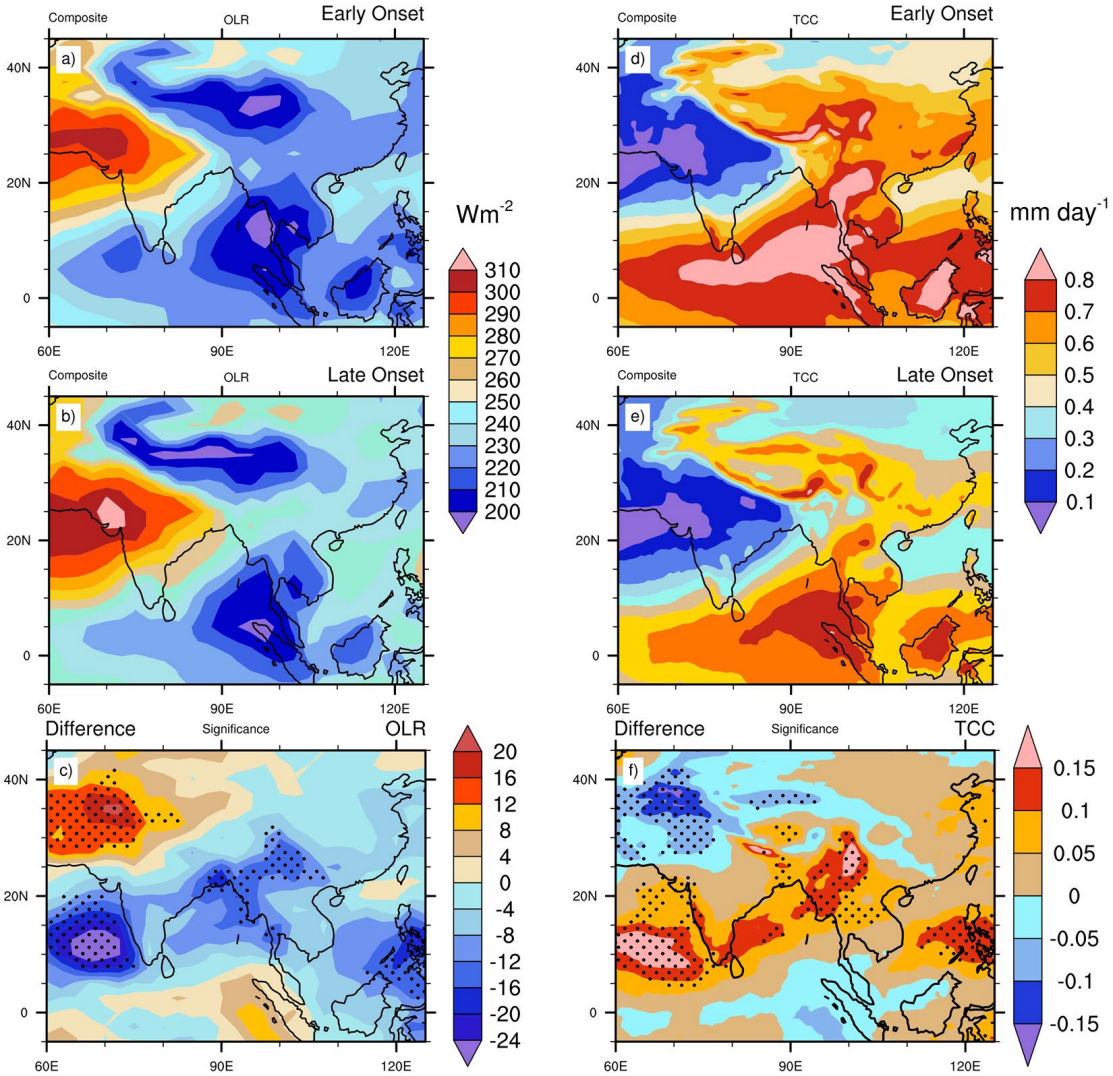
Composite mean May OLR (Wm^−2^) and Total Cloud Cover (mm/day) for Early, Late onset years and its difference (left and right side respectively). Dots indicate 95% confidence level based on a two tailed *t-*test. The maps in the figure are generated using NCL software [The NCAR Command Language (Version 6.6.2) [Software]. (2019). Boulder, Colorado: UCAR/NCAR/CISL/TDD. http://dx.doi.org/10.5065/D6WD3XH5].

**Figure 2:**
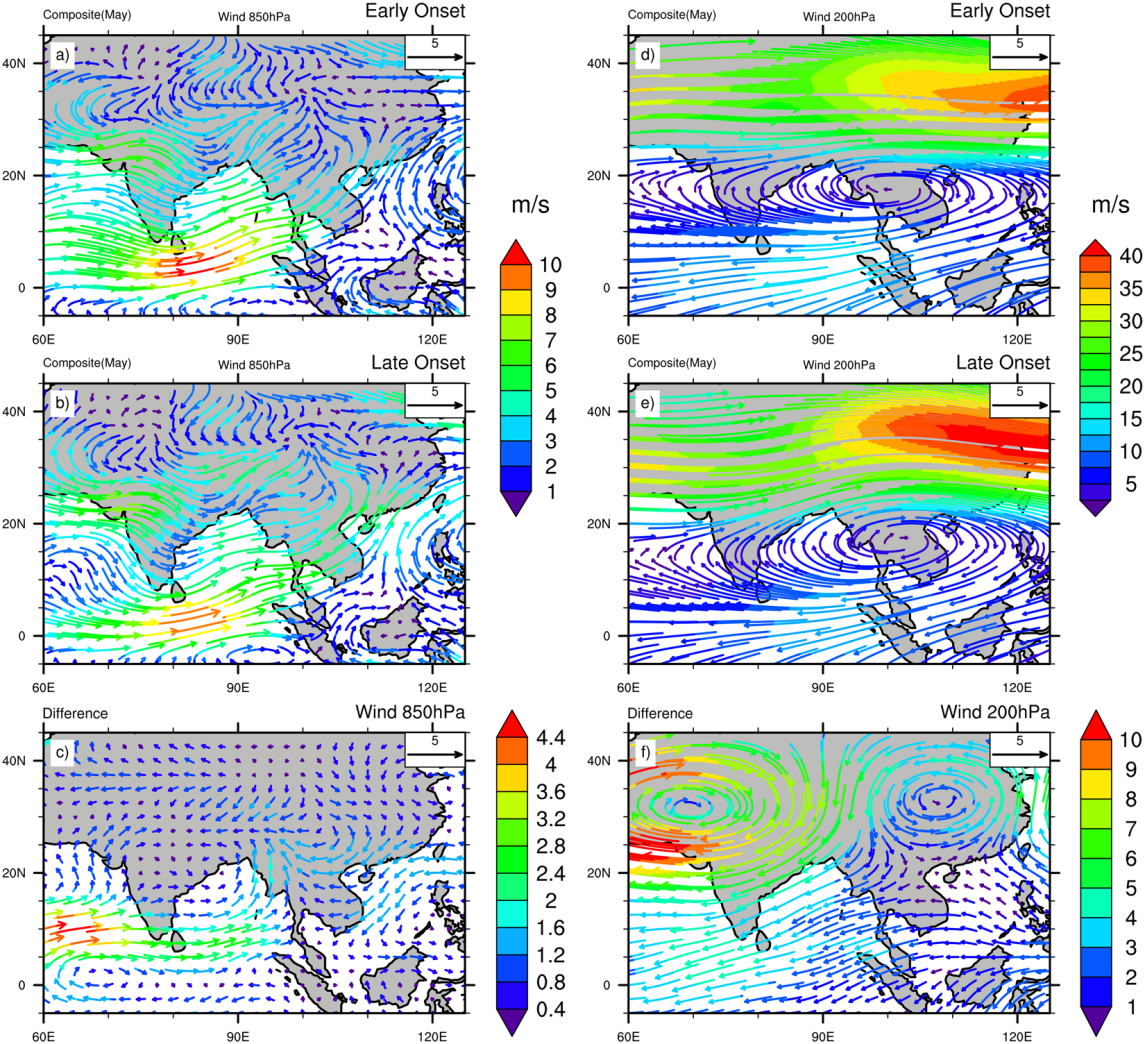
Composite mean May wind at 850 and 200 hPa (ms^−1^) for Early, Late onset years and its difference (left and right side respectively). The maps in the figure are generated using NCL software [The NCAR Command Language (Version 6.6.2) [Software]. (2019). Boulder, Colorado: UCAR/NCAR/CISL/TDD. http://dx.doi.org/10.5065/D6WD3XH5].

To examine the remote influences on the variability of MOK, composite analyses during the month of May are employed for the early and late onset years. The monthly mean composite analysis of Outgoing Long wave Radiation (OLR) and total cloud cover for the early, late onset and its differences (Early-Late onset) are shown in Fig. 1. The OLR field (Fig. 1a–c) shows that a band of convection (low value of OLR) develops in the eastern BOB and TP during MOK. During the early onset, the convection bands over the AS and eastern BOB are stronger than the late onset (Fig. 1c). Along the band of convection (from southern AS to eastern BOB), the entire region remains significantly cloudy in the early onset (Fig. 1d). The composite difference in total cloud cover is significant over almost the entire AS region (Fig. 1f). The composite mean wind at 850 hPa and 200 hPa for the early and late onset years are shown in Fig. 2. The cross equatorial LLJ over the AS and BOB are more intense in the early onset years, while in the late onset years, the strength of wind at 850 hPa over the south east Asia (SEA), South China Sea (SCS), south China and the eastern TP are stronger (Fig. 2a–c). The largest difference of about 4.4 ms^−1^ is noticed over the southern AS (Fig. 2c). On the other hand, during the late onset, the Sub Tropical Jet stream (STJ) at 200 hPa is stronger over the east of TP and China as compared to the early onset (Fig. 2d–f). The South Asian High (SAH) centers over SEA’s landmass for both the onsets, but a prominent ridge forms over the eastern TP in the STJ during late onset. To examine the changes in SST, a composite of SST during the early onset, late onset and its difference is shown in Supplementary Fig. S1a–c. During the late onset, the warmer SST dominates in the NIO region especially in the BOB and AS, and colder SST prevails in the extreme South Indian Ocean (SIO) adjacent to the Mascarene High (MH, ~30^0^–35^0^S, Supplementary Fig. S1b). These features are in agreement with Sabeerali *et al*.^11^, where they argued that the delay in monsoon onset is linked with warmer Indian Ocean SST. The composite difference of the SSTs between the early and late onset shows a significant decrease in SST in the NIO region during the early onset years (Supplementary Fig. S1c). Furthermore, we also investigate the composite pattern of Sea Level Pressure (SLP) for the early and late onset which is shown in Supplementary Fig. S1d–f. In general, during the pre-monsoon season, due to strong land-sea thermal contrast an intense low pressure builds up over the northern Indian subcontinent, while high pressure forms over SIO (especially around the Madagascar Island). It is observed that a region of significant lower SLP over the AS and Indian subcontinent persists in the early onset, while during the late onset, this band of lower SLP is significantly extended towards the southern China (Supplementary Fig. S1f). Also, the strength of the MH appears to enhance over the SIO during the early onset.

### Daily composite evolution

**Figure 3:**
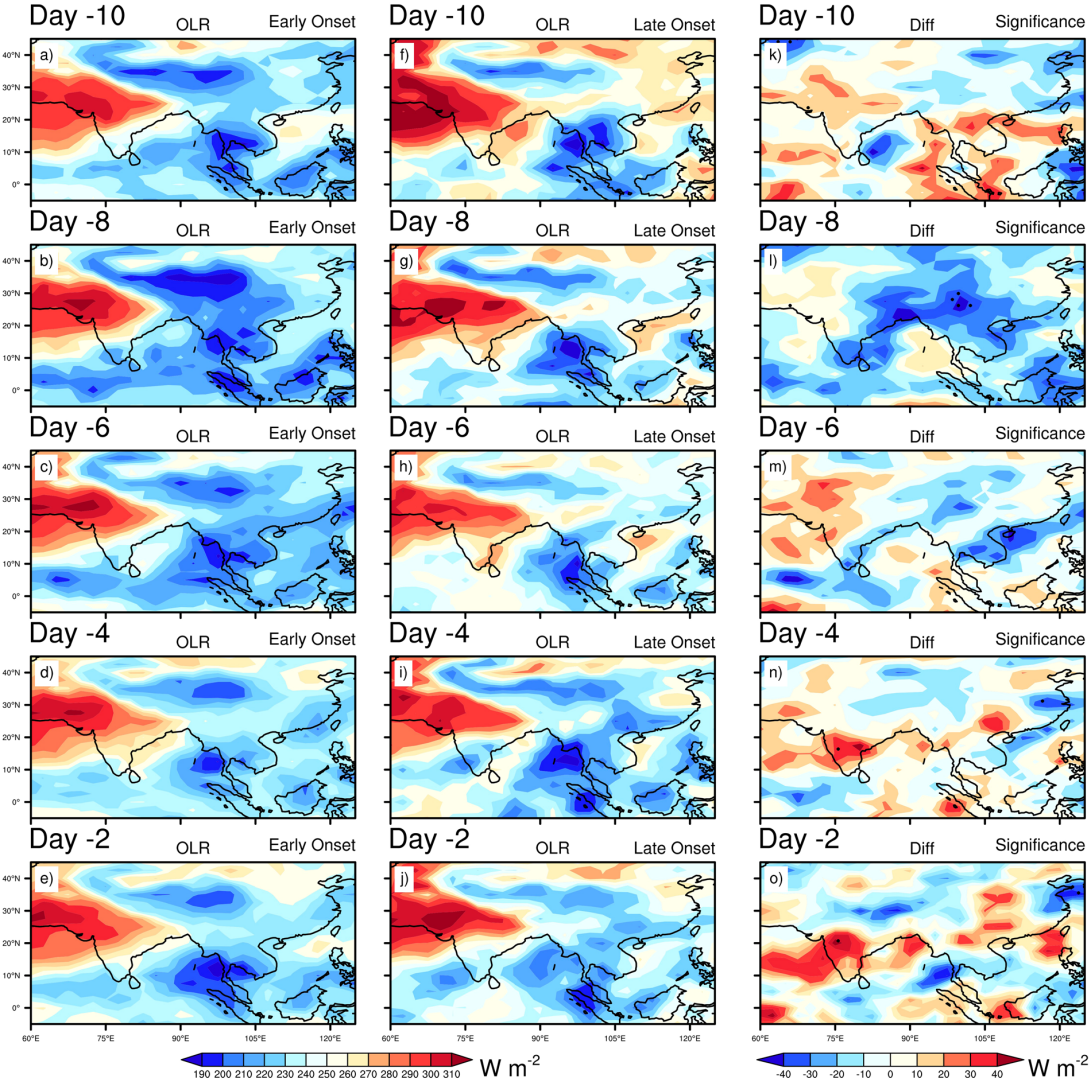
Composite mean daily evolution of OLR (Wm^−2^) from 10 days (Day -10) prior to the onset to 2 days before the onset (Day -2) for Early (extreme left), Late onset (middle) and its differences (extreme right). Dots indicate 95% confidence level based on a two tailed *t*-test. The maps in the figure are generated using NCL software [The NCAR Command Language (Version 6.6.2) [Software]. (2019). Boulder, Colorado: UCAR/NCAR/CISL/TDD. http://dx.doi.org/10.5065/D6WD3XH5].

**Figure 4:**
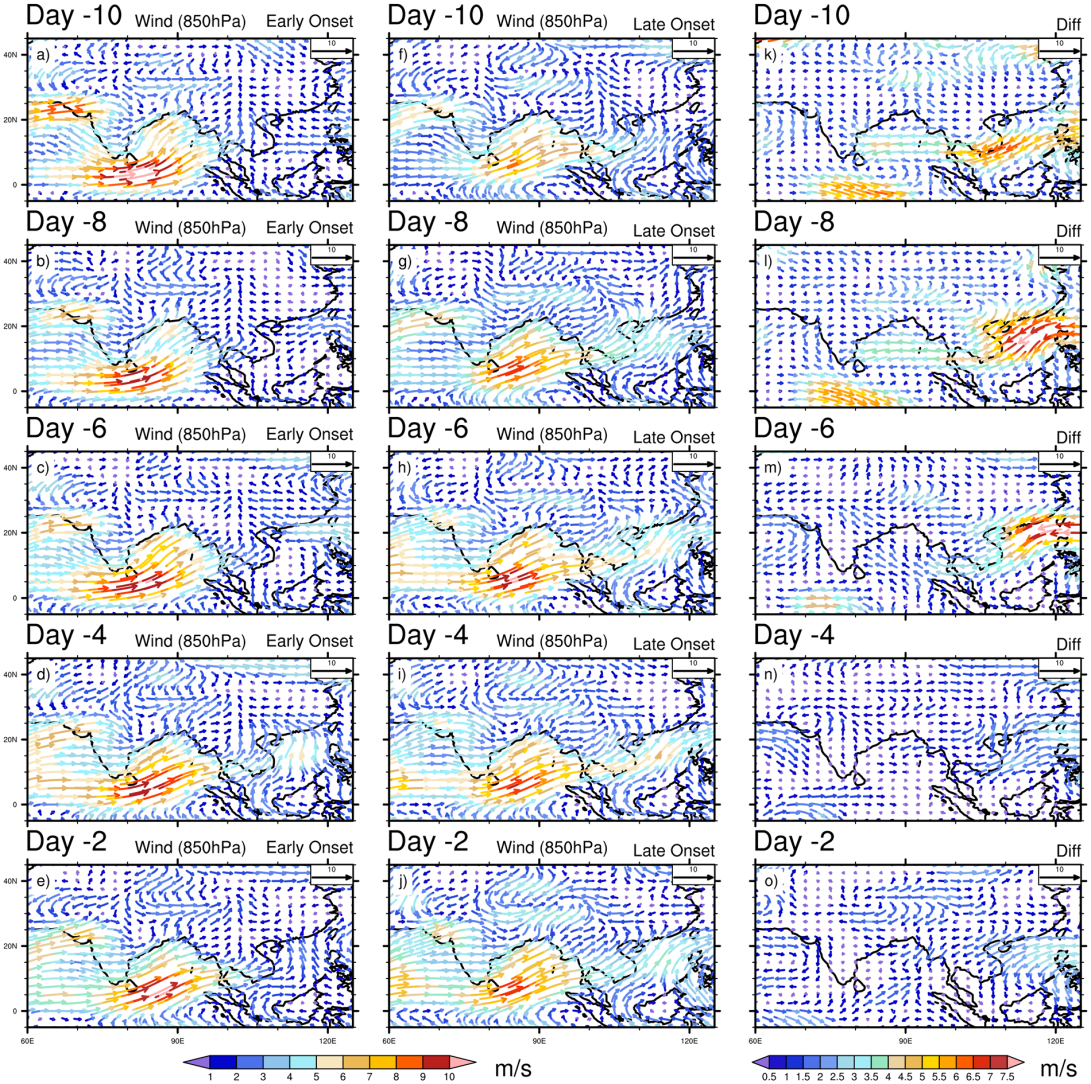
Composite mean daily evolution of Wind (ms^−1^) at 850 hPa from 10 days (Day -10) prior to the onset to 2 days before the onset (Day -2) for Early (extreme left), Late onset (middle) and its differences (extreme right). The maps in the figure are generated using NCL software [The NCAR Command Language (Version 6.6.2) [Software]. (2019). Boulder, Colorado: UCAR/NCAR/CISL/TDD. http://dx.doi.org/10.5065/D6WD3XH5].

**Figure 5:**
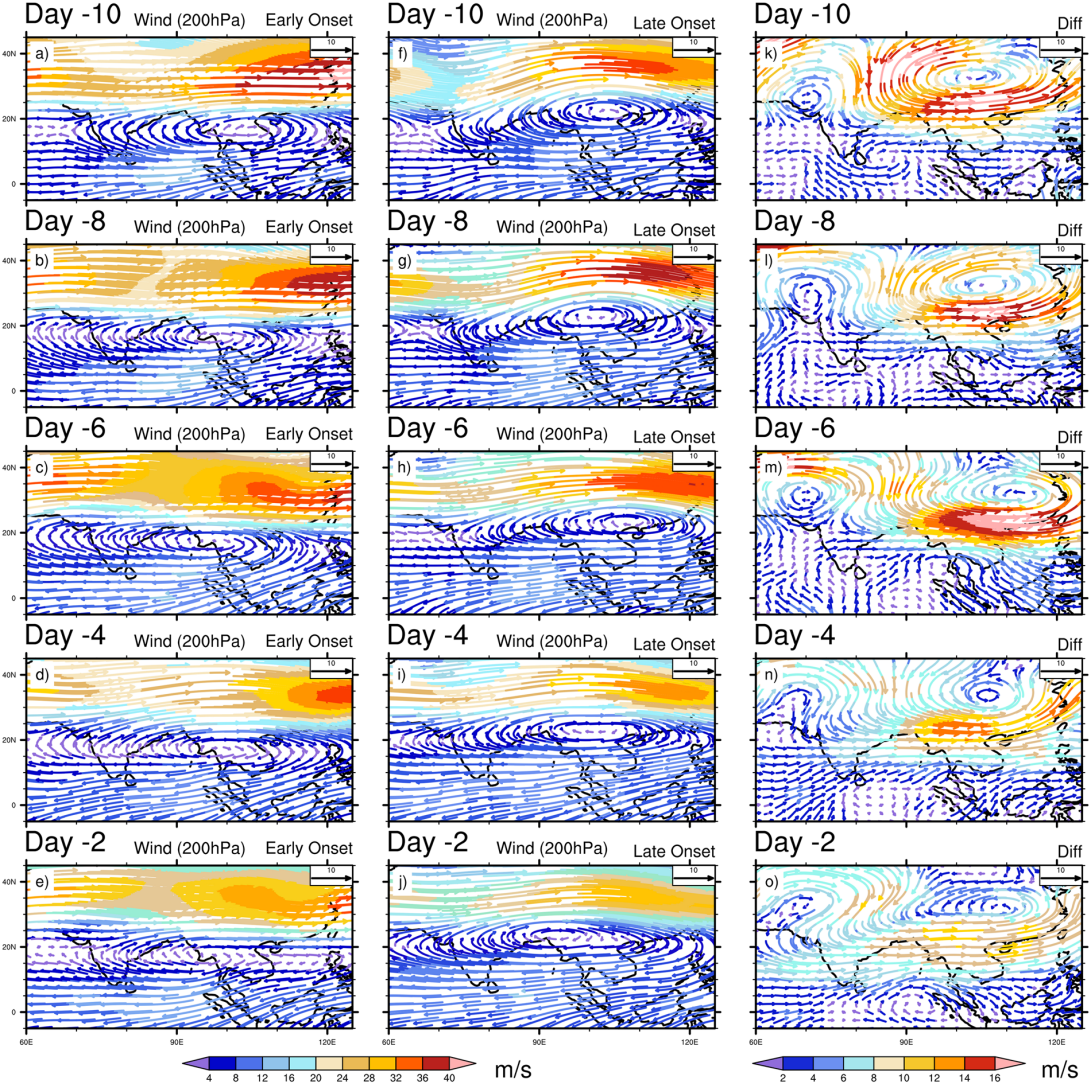
Composite mean daily evolution of Wind (ms^−1^) at 200 hPa from 10 days (Day -10) prior to the onset to 2 days before the onset (Day -2) for Early (extreme left), Late onset (middle) and its differences (extreme right). The maps in the figure are generated using NCL software [The NCAR Command Language (Version 6.6.2) [Software]. (2019). Boulder, Colorado: UCAR/NCAR/CISL/TDD. http://dx.doi.org/10.5065/D6WD3XH5].

To further examine the differences in the mean daily evolution pattern prior to the early and late MOK, we investigate the composite daily mean OLR, Omega (vertical velocity), winds (850 hPa, 200 hPa), horizontal moisture convergence flux, precipitation, SST, SLP, Surface Air Temperature (SAT, i.e. air temperature at 1000 hPa) and temperature advection at 850 hPa respectively. For daily evolution, we start the analysis 10 days prior to the onset dates, and investigate at days 10, 8, 6, 4, 2 (Hereafter, read as Day -10, Day -8, Day -6, Day -4, Day -2 respectively) before the corresponding onset dates. Figure 3 shows the composite of OLR in the early, late onset and its difference. At Day -10, an active convection center is located over the eastern BOB, SEA’s landmass, TP and south China during MOK. Comparatively a low OLR appears during the early onset years over the western BOB and eastern TP, while in the late onset, more convective activities are confined over the eastern BOB (Fig. 3k). These active convective centers get stronger during the early and late onset at Day -8 (Fig. 3b,g), but the composite difference shows that during the early onset years, convection grows stronger over the eastern TP, south China, south-east to north-eastern part of India except the eastern BOB, where more convective activities are confined during the late onset (Fig. 3f). The convection is the strongest at Day -8, and from Day -6 to -2 the convective activities gradually weaken and confine mainly over the eastern BOB and TP for both the early and late onset (Fig. 3c,h,m,d,i,n,e,j,o). In the daily evolution of OLR, the convective centers are more active in the early onset over the southern AS, along the path of LLJ and TP. Moreover, it is worth mentioning that during the entire late onset’s evolution period, the center of convection remains active over the eastern BOB as compare to the early onset. The evolution of vertical velocity (Omega at 925 hPa) is presented in Supplementary Fig. S2. It is observed that across the entire evolution window (Day -10 to -2) more continuous updrafts (negative value of Omega) can be seen over the west, south east India, BOB, TP during the early onset (Supplementary Fig. S2a–e). We can observe a sharp contrast in updrafts during the late onset period, except some parts of the eastern India and eastern part of TP; the downdrafts are prominent elsewhere (Supplementary Fig. S2f–j). During the early onset, a deep convection over the BOB plays a key role to enhance the updraft. However, it is also noted that significant changes in Omega (Day -10, -8, -2) are found over the eastern India extending up to the eastern TP and southern China (Supplementary Fig. S2k–o), which explain more updrafts over there in the late onset. The wind at 850 hPa is shown in Fig. 4, which presents an intense cross equatorial LLJ over BOB during the early onset for the entire evolution period (Fig. 4a–j). The maximum wind speed is observed at Day -6 both for the early and late onset (Fig. 4c,h). Overall, the wind speed shows a gradual weakening trend from Day -10 to -2. The major difference between the two categories lies over the SEA and SCS (Fig. 4k–o), where across the entire evolution, winds blowing from the BOB towards the SEA, SCS, eastern TP and southern China become stronger during the late onset. Therefore, this strong wind may strengthen the large-scale moisture convergence over the eastern TP and southern China during the late onset. The composite mean of wind at 200 hPa is also shown in Fig. 5. The STJ over TP and the SAH centered over the SEA’s landmass are the two prominent features in the upper air map shown in Fig. 5. The strongest STJ is found at Day -8 both for the early and late onset (Fig. 5b,g), however, for the entire evolution period, the STJ is consistently stronger during the early onset as against the late onset. During the late onset, a prominent ridge forms over the east of TP and China accompanied with the stronger SAH (upper air divergence, Fig. 5f–j). The center of SAH lies consistently over the north-eastern BOB and adjoining Myanmar during the early onset, while during the late onset, the center moves towards the south China due to stronger low-level convergence over the eastern TP and southern China. Therefore, during the late onset years, the strengthening of this convergence zone over the eastern TP brings more moisture to converge over the TP and adjacent region, which is clearly shown in moisture convergence plot (Supplementary Fig. S3). It is known that the intensity of SAH depends on the distribution of heterogeneous diabatic heating over the TP and the ASM area (Wu and Liu 2003^22^; Wu *et al*.^23^). During the entire evolution period both for the early and late onset, a significant amount of moisture converges over the TP. However, in the early onset, more moisture converge over the India especially in the eastern and north-eastern part of India, where the difference is significant at Day -8, -6, -4 (Supplementary Fig. S3l,m,n). The precipitation during the ISM onset primarily confines over the eastern BOB, adjacent Myanmar and along the path of cross-equatorial LLJ over NIO (Supplementary Fig. S4). It is clear that precipitation over the southern BOB is considerably higher during the early onset (Supplementary Fig. S3k–o). During late onset, the region of higher precipitation over the north-eastern BOB collocates with the region of higher convective activities, and higher precipitation over the eastern TP to southern China is also collocated with the stronger convergence zone during the entire evolution period.

**Figure 6:**
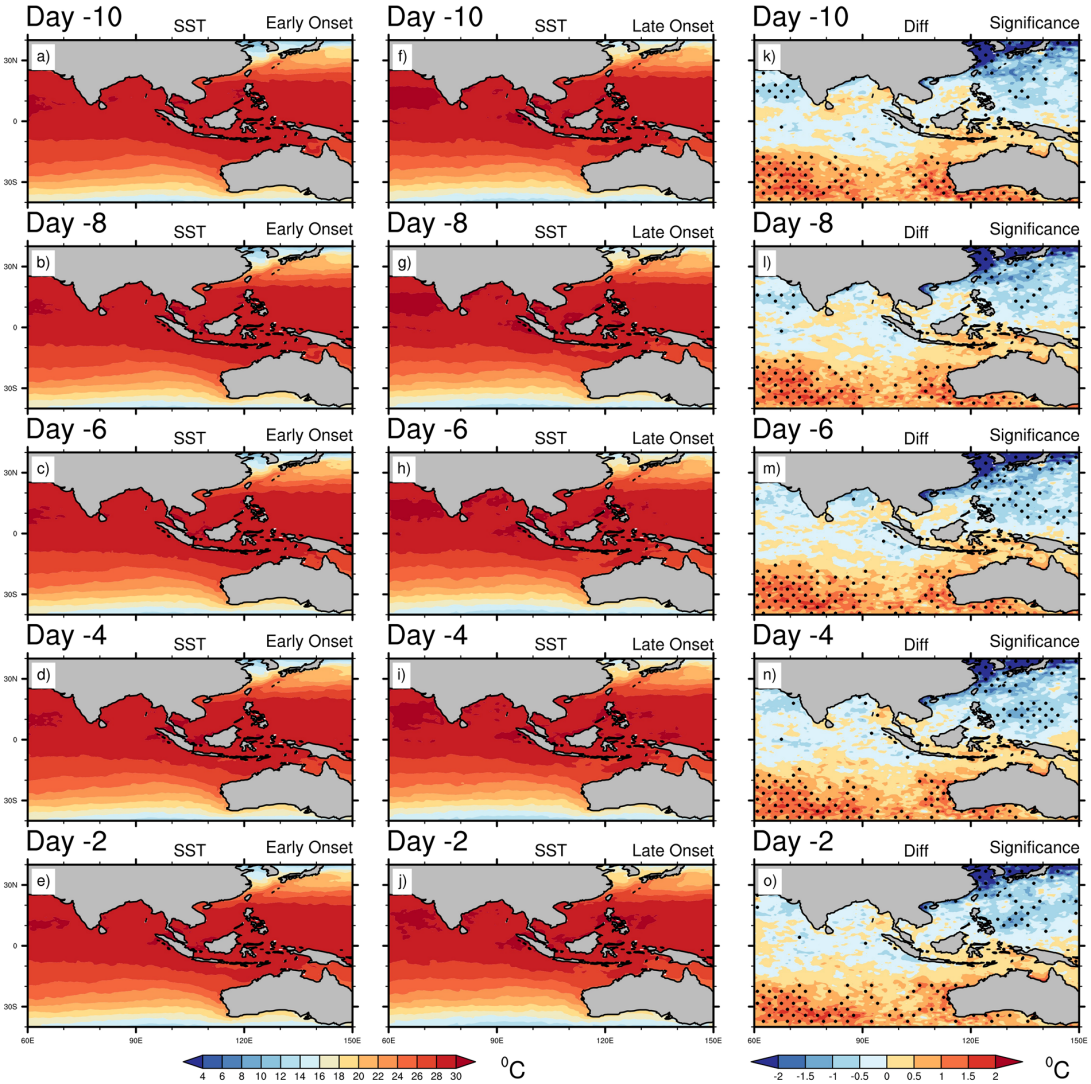
Composite mean daily evolution of SST (°C) from 10 days (Day -10) prior to the onset to 2 days before the onset (Day -2) for Early (extreme left), Late onset (middle) and its differences (extreme right). Dots indicate 95% confidence level based on a two tailed *t*-test. The maps in the figure are generated using NCL software [The NCAR Command Language (Version 6.6.2) [Software]. (2019). Boulder, Colorado: UCAR/NCAR/CISL/TDD. http://dx.doi.org/10.5065/D6WD3XH5].

**Figure 7:**
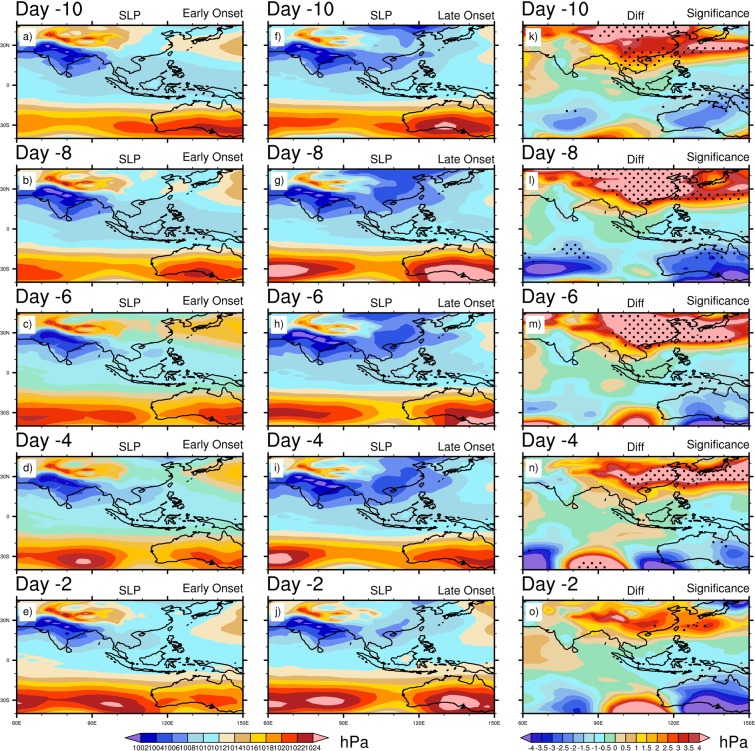
Composite mean daily evolution of SLP (hPa) from 10 days (Day -10) prior to the onset to 2 days before the onset (Day -2) for Early (extreme left), Late onset (middle) and its differences (extreme right). Dots indicate 95% confidence level based on a two tailed *t*-test. The maps in the figure are generated using NCL software [The NCAR Command Language (Version 6.6.2) [Software]. (2019). Boulder, Colorado: UCAR/NCAR/CISL/TDD. http://dx.doi.org/10.5065/D6WD3XH5].

**Figure 8:**
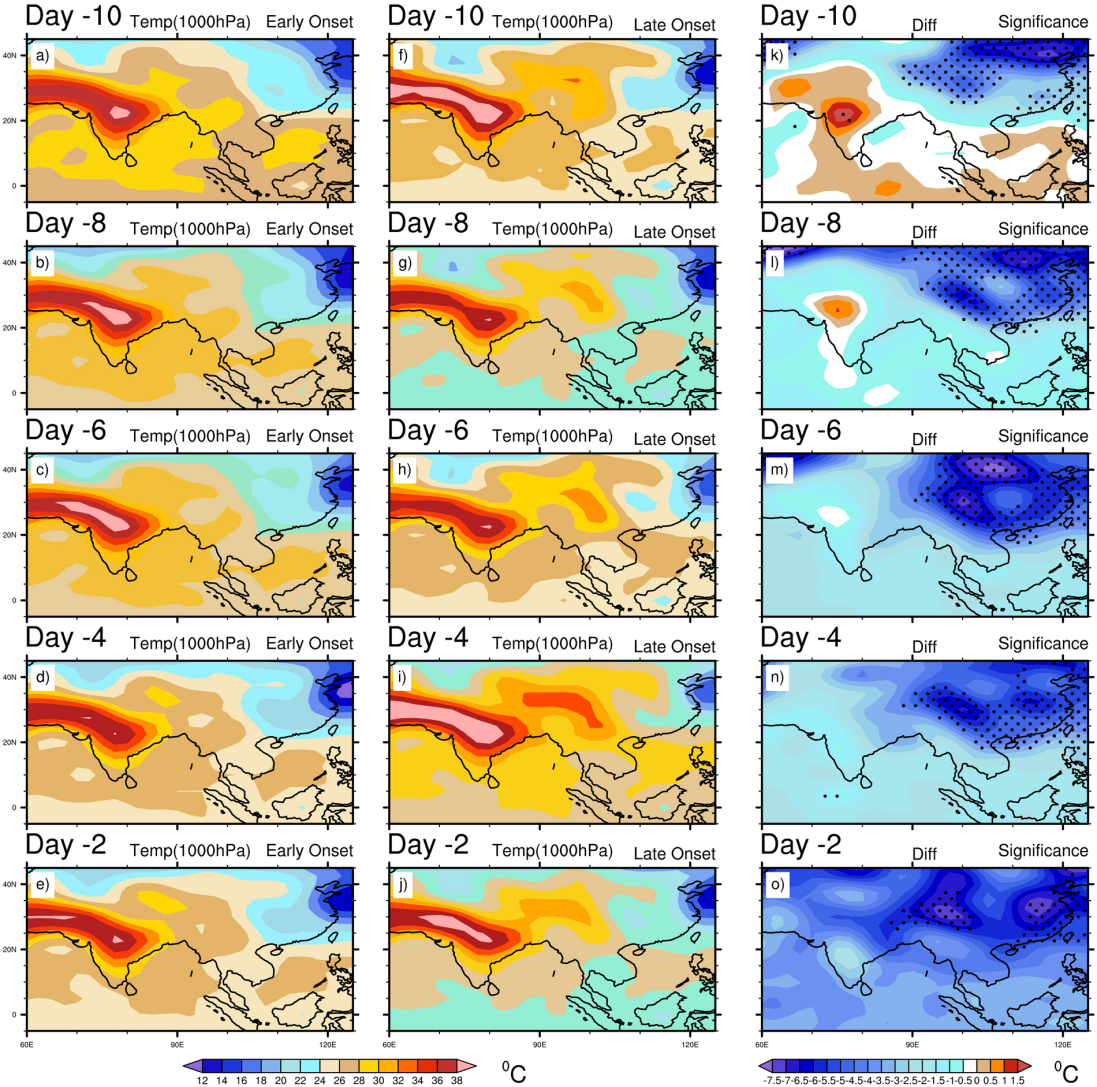
Composite mean daily evolution of surface air temperature (°C) at 1000 hPa from 10 days (Day -10) prior to the onset to 2 days before the onset (Day -2) for Early (extreme left), Late onset (middle) and its differences (extreme right). Dots indicate 95% confidence level based on a two tailed *t*-test. The maps in the figure are generated using NCL software [The NCAR Command Language (Version 6.6.2) [Software]. (2019). Boulder, Colorado: UCAR/NCAR/CISL/TDD. http://dx.doi.org/10.5065/D6WD3XH5].

Therefore, the above composite analyses produce some distinctive circulation features during the early and late onset years. The late onset is associated with more convection over the eastern BOB, a significant amount of updraft over the eastern TP and southern China, whereas a stronger convergence and upper air divergence (SAH) over the eastern TP and southern China. The synoptic features in the late onset are also included with stronger wind at 850 hPa blowing from the BOB to the eastern TP and southern China, higher moisture convergence and precipitation over eastern TP. On the contrary, the early onset features include deep convection over the southern BOB and southern AS, stronger cross-equatorial LLJ over the tropical Indian Ocean, more updrafts over the south, eastern India, BOB, TP and SEA, more moisture convergence over the India, AS and BOB and higher precipitation over the BOB, which are usually regarded as the normal meteorological features during normal MOK. These changes in mean circulation during the late onset warrant further investigation and therefore, we analyze the daily evolution of SST, SLP and SAT. Figure 6 shows the composite mean daily evolution of SST for the early, late onset and its difference. It is seen that the NIO becomes warmer, and the SIO and adjacent to the MH remain colder consistently during the entire evolution period. The SST in the SIO becomes significantly colder during the late onset in comparison to the early onset (Fig. 6f–j). In the NIO region, although the difference is not significant during the early onset years, but the ocean remains warmer along the path of LLJ or from the southern AS to BOB. It is worth to mention that the late onset is associated with the significant higher SST over the North Pacific Ocean (~30^0^N) during its entire evolution, which may trigger the stationary wave train reaching into the central Asia and thus, may generate a warm temperature anomaly over the China and adjacent region. During the late onset, the lower SST over SIO is reflected in the composite SLP analysis (Fig. 7). The significant changes in SLP between the early and late onset years are associated with lower SLP over the eastern TP to southern China and higher SLP over the western MH during the late onset years (Fig. 7k–o). At Day -8, the maximum amount of difference between the early and late onset is found (Fig. 7b,g,l). In the late onset, the colder SIO is perfectly collocated with the region of higher SLP. Thus, the anomalous changes of SLP over the east Asian land-mass play a major role in the onset processes. In order to investigate the anomalous changes of SLP, we analyze the SAT in Fig. 8, which shows a significant change across the evolution period over the eastern TP to central and southern China (Fig. 8k–o). Although, the Indian landmass, AS, and BOB experiences a warmer SAT during the early onset, but the difference remain insignificant. On the contrary, the SAT is significantly higher over the eastern TP to central and southern China’s land-mass during the late onset (Fig. 8f–j). In the difference plot, two centers of high temperature are prominent over the eastern TP, and another one moves from the northern China to south-eastern China during the evolution time period. The SAT from the north China (at Day -10) advects gradually towards the eastern and southern TP (as seen at Day -8, -6, -4), and at Day -2 the temperature advects further eastward and north-eastern part of India (Fig. 8o, also see temperature advection plot Supplementary Fig. S5). From the above analysis, It can be clearly seen that the advection of SAT from the north of China’s landmass to the southern-eastern TP plays the key driving role to control and modulate the MOK process and its variability.

### Analysis of an extreme early and late onset

To confirm the robustness of our study, we also perform a similar analysis for the earliest (12^th^ May, 1999) and latest (11^th^ June, 2012) MOK years from the list given in Table 1. The monthly mean May composite analysis of OLR and total cloud cover for the earliest onset, latest onset and its differences are shown in Supplementary Fig. S6. During the earliest onset, the convection bands located over the AS, the Western Ghat region, eastern BOB, southern TP and Myanmar, while the convective activities mostly confined over the south-eastern BOB during the latest onset (Supplementary Fig. S6b). It is clear that large convection were occurred over the AS along the Western Ghat region during the earliest onset as compared to the latest onset (Supplementary Fig. S6c). Similarly, the southern AS region was cloudier in the earliest onset (Supplementary Fig. S6f). The cross equatorial LLJ over the AS and BOB was stronger in the earliest onset year, while during the latest onset year, the wind at 850 hPa strengthened over SEA, SCS and eastern TP (Supplementary Fig. S7a–c). On the other hand, the SAH centered over the Myanmar adjacent to the north-east BOB during the earliest onset year, while in the latest onset year, the center of SAH moved towards the south-east China, and the STJ was relatively stronger over the eastern China’s landmass by forming a ridge (Supplementary Fig. S7d–f).

Also, we investigate the daily evolution of OLR, wind at 850 hPa and 200 hPa, SST, SLP and SAT prior to the earliest and latest onset year. In OLR, it can be seen that the convection mostly confined over the southern AS and head BOB (along the coast of Western Ghat) from Day -6 onward during the earliest onset, while the band of convection stationed over the eastern BOB, eastern equatorial Indian Ocean and SCS during entire period of the latest onset event (Supplementary Fig. S8). The core of LLJ (wind at 850 hPa) is seen mostly over the AS to the south BOB and moving towards SCS during the latest onset evolution, whereas the LLJ was strengthened towards the head BOB during the earliest onset event (Supplementary Fig. S9). It is clear that LLJ was stronger over SCS, AS during the latest onset year (Supplementary Fig. S9k–o). In wind at 200 hPa, it is well observed that the STJ was more intense during the earliest onset, and the eastern China was dominated by the strongest STJ (Supplementary Fig. S10). The strength of STJ was weakened gradually from Day -10 to -2 prior to the onset for both the cases. The SAH initially centered over the adjacent landmass of the BOB at Day -10, then it slowly moved eastward and stationed over south China during the latest onset case. On the other hand, during the earliest onset, the SAH which initially centered over the SEA, moved slightly westward and helped the convection to grow over the BOB. The pattern of SST evolution is found to be similar with that in the earlier composite SST analysis (Supplementary Fig. S11), where warmer SST prevailed in the north Pacific and SIO during the latest and earliest onset year respectively. The pattern of SLP evolution was approximately similar to the earlier composite analyses, where we can see that Indian landmass was dominated by the lower SLP, and the MH at 30^0^S was strengthened during entire period of the earliest onset case (Supplementary Fig. S12). On the contrary, during entire period of the latest onset, the band of low SLP over the India was extended up-to the north-eastern China and the north-western Pacific Ocean, and the MH was slightly moved west-ward (Supplementary Fig. S12f–j). Finally, the higher SAT is seen over the China and India (only at Day -10 and -6) during the latest onset year, while during the earliest onset year, Indian Ocean was warmer at Day -8, -4 and -2 (Supplementary Fig. S13). Overall, similar type of features as seen above are observed in the extreme event analyses.

### Daily evolution of MOKs without ENSO years

It is already discussed in introduction that delays in MOK are strongly associated with the El Niňo^2^. The difference in SST during ENSO induces the variability in monsoon onset by affecting the movement of the ITCZ^3^. Therefore, to examine the pattern of the onset evolution during the non-ENSO years, we analyze a new set of MOK list by excluding those MOK years which are associated with El Niño and La Niña. Out of 14 early MOKs, 5 and 9 onsets are associated with La Niňa and El Niňo (over Niňo-3.4 region) years, respectively (Table 1, Supplementary Fig. S14). It is also noted that since 1979, all the La Niňa years are associated with the early MOKs. Here, we only discuss wind at 850 hPa, 200 hPa, SLP and SAT respectively. The wind at 850 hPa is shown in Supplementary Fig. S15, which also reflects an intense cross equatorial LLJ over the BOB and equatorial Indian ocean during early onset evolution period. While during late onset evolution, the LLJ moves towards the SEA, SCS, eastern TP and southern China. As observed before, the wind at 200 hPa also shows that the STJ is consistently stronger during the early onset as compared with the late onset (Supplementary Fig. S16). During the late onset, a prominent ridge forms over the east of TP and China accompanied with the stronger SAH centered over the southern China. The lower SLP over the eastern TP to eastern China and higher SLP over the western MH are associated with the late onset years, which in turn play a major role during the onset processes (Supplementary Fig. S17). Finally, evolution of SAT presents a significant change over the eastern TP to the central and southern China during the late onset years (Supplementary Fig. S18). The SAT is significantly higher over the eastern TP to the central and southern China’s land-mass during the late onset years (Supplementary Fig. S18f–j). Therefore, as already observed above, the SAT from the north China (as Day -10) advects gradually towards the eastern TP and China during the late onset years. Thus, here, these similar type of circulation patterns during the non ENSO years further prove the key role of the SAT over the east Asia in the onset modulating processes.

### Potential factors responsible for late MOKs

The late onset of monsoon is one of the most undesired events in context of the ISM system. Several authors have tried to explain the possible reasons behind the early and late onsets (mentioned in the introduction). It is well known that due to a strong land-sea thermal contrast during the pre-monsoon season, intense low and high SLP built up over the Indian subcontinent and the Madagascar Island (in the form of MH) respectively. These intense thermal and pressure gradient between the SIO and Indian subcontinent strengthen the monsoonal jet (cross-equatorial LLJ) with abundant of moisture influx from the warm Indian ocean to the Indian subcontinent, which eventually triggers the deep convection and heavy rainfall over ocean and land. Here, based on the composite analyses, it’s observed that the late onset is clearly linked with the surface warming over the northern, southern China and the eastern TP, which may be the attribution of the warmer north Pacific. During the late onset years, the extension of low SLP over the east Asia (eastern TP, south-east-central China, SCS, and Sea of Japan) due to anomalous high SAT over the eastern TP, southern China and the adjacent region strengthen the south-westerly LLJ over the east Asia from the BOB. The SAH is probably modulated by the anomalous warming, and it moves north-eastward and intensifies over the southern China, whereas during the BOB monsoon onset, the center of SAH normally moves north-westward. The surface warming also strengthens the wind convergence over the eastern TP and southern China, and therefore, increases the moisture influx from the BOB. The lack of sufficient moisture availability over BOB and peninsular India, weaker updrafts over ISM region and weaker LLJ over NIO are the potential key factors behind the late MOKs. The background atmospheric conditions for the early and late MOK and the role of east Asia are shown in the schematic diagram (Supplementary Fig. S19).

## Summary and Conclusion

In this study, we investigate the physical mechanism and probable meteorological drivers behind the late MOK process. In order to carry out the investigation, we select 14 early and 9 late onset years (5 days or more after and before the normal onset date as ‘late’ and ‘early’ onset year respectively). The 1^st^ June is chosen as normal MOK date as determined by the IMD, and the list of early and late onset dates are tabulated in Table 1. Then, we perform the May composite analyses for the early and late onset years. Next, to examine the differences in daily evolution between early and late MOK, a composite mean daily evolution (from -10 days to -2 days prior to the onset) of several meteorological variables are investigated. To have robustness of the study, we perform a comparative analysis by choosing an extreme early and late MOK (12^th^ May, 1999 and 11^th^ June, 2012 are chosen as the earliest and latest MOKs respectively). Also, to examine the pattern of onset evolution during the non ENSO years, we analyze a new set of MOK list by excluding those MOK years which are associated with El Niño and La Niña. The entire results can be summarized as follows:

**Table 1:** List of Early and Late onset dates with La Niña and El Niño (Niño-3.4 region) years.

Sr. No	Early Onset Dates	Late Onset Dates
1.	1981-05-25	1979-06-09
2.	1985-05-22	1983-06-08 (**El Niño**)
3.	1989-05-24 (**La Niña**)	1986-06-06
4.	1990-05-15	1997-06-09
5.	1994-05-26	2005-06-08
6.	1999-05-12 (**La Niña**)	2007-06-09
7.	2000-05-14 (**La Niña**)	2012-06-11
8.	2001-05-18	2014-06-06
9.	2002-05-23	2016-06-08 (**El Niño**)
10.	2004-05-13	
11.	2006-05-21	
12.	2008-05-25 (**La Niña**)	
13.	2011-05-26 (**La Niña**)	
14.	2013-05-22	

1. The composite mean May OLR suggests that during MOK, the convection (low values of OLR) grow in the eastern BOB and southern TP. But during the early onset, the convection over the AS and eastern BOB are stronger than the late onset. In the daily evolution, the convective centers grow more in the early onset over the southern AS, at the path of LLJ and the TP, while during the late onset’s entire evolution period, the center of convection consistently remains more active over the eastern BOB as compared to the early onset evolution.

2. In entire daily evolution period, a consistent updraft can be seen over the Indian subcontinent except a band of downdraft zone over the northern India during the early onset, whereas during late onset, the updrafts shift towards the east of India to the eastern TP and southern China.

3. In the May composite, the cross equatorial LLJ over the AS and BOB is observed as stronger in early onset, while in the late onset, the stronger wind at 850 hPa over SEA, SCS and eastern TP is observed. Also, the STJ over the eastern TP is more intense in the early onset, but the upper-air anticyclonic system associated with the STJ is found as stronger with a ridge forming over the eastern China during the late onset. In the daily evolution, the LLJ flows significantly from the BOB towards the SEA, SCS, southern China to eastern TP, thus strengthens the convergence zone over the southern China and eastern TP during the late onset. The strongest STJ is found at Day -8 for both the early and late onset. The center of the SAH lies consistently over the north-eastern BOB and adjoining Myanmar during the early onset, while in the late onset, the SAH center moves towards the south China’s landmass due to the presence of strong low-level convergence over there.

4. The strengthening of this convergence zone over the eastern TP helps to attract more moisture from the BOB during the late onset. During the whole onset evolution, a significant amount of moisture is seen to converge over the TP for both the early and late onset due to presence of this stronger convergence zone. However, the early onset consistently show with more moisture convergence over the India, especially over the east, north-east India. In the daily evolution of Precipitation, it is also shown that the precipitation over the southern BOB is considerably higher during the early onset except Day -4. The region of higher precipitation over the north-eastern BOB collocates with higher convective activities, and consistent more precipitation over the eastern TP to the southern China (significant at Day -4) are also co-centered with the strong convergence zone during the entire evolution in the late onset.

5. Based on May composite analysis, the late MOK are associated with warmer SST in the NIO especially in the BOB, AS and colder SST in the extreme SIO near to the MH (~35^0^S-30^0^S). Similar type of features in SST are also seen during daily evolution, i.e warm SST in NIO and cold SST in the extreme SIO or near to the MH, while SST at the MH remains significantly lower during the late onset years for the entire evolution period. Also, the late onset is associated with the significant warmer SST over the north Pacific Ocean (~30^0^N), which may trigger the stationary wave train reaching into central Asia and thus, generates a warm anomaly over the China and adjacent region.

6. The composite SLP of May shows comparatively lower value during the early onset over the AS and west-central-eastern India and higher value over the eastern TP and north-west China, and the strength of the MH enhances insignificantly during the late onset years over the central of extreme SIO. In the SLP daily evolution, it is found that the significant changes between the early and late onset are associated with lower SLP over the eastern TP to central and southern China’s landmass, and a higher SLP is centered over west of 30^o^S or western MH region during the late onset. At Day -8, the largest significant difference is found, where intense low SLP over the eastern TP to southern China and higher SLP over the western MH during late onset can be observed.

7. In order to investigate the anomalous changes of SLP, we also address SAT. The SAT increases significantly in the late onset years, stretching from the northern China to the eastern TP. Two centers of higher temperature can be seen from the difference plot; one is near the eastern TP and another one is moving from the central China to the south-eastern China during the evolution. The SAT from the northern China (as Day -10) gradually advects towards the eastern and southern TP (as seen at Day -8, -6, -4), and at Day -2 SAT advects further towards the east and north-east of India. The SAH is probably modulated by this anomalous warming and thus, it moves north-eastward rather than normal north-westwards.

From the above analysis, it is clear that SAT advection from the north of China’s landmass to the south and east of TP plays a driving role to modulate the MOK processes. The extension of low SLP over the east Asia (eastern TP, south-east-central China, SCS, Sea of Japan) due to anomalous high SAT over the eastern TP and adjacent region draws the south-westerly LLJ from the BOB to the east Asia during the late onset. Hence, it strengthens the moisture convergence over the eastern TP. Therefore, the lack of moisture over the BOB and India’s eastern land-mass and comparatively weaker LLJ over the eastern BOB are the probable causes behind the late ISM onset. In a nut shell, the anomalous high SAT over the eastern TP extending up-to central and eastern China makes an anomalous low SLP over there resulting diversion of moisture transport from the BOB towards the east Asia, which pushes the MOK to be delayed. Thereby, the southward SAT advection from the north China towards the TP, north-eastward movement of SAH and warming over the North Pacific Ocean are playing some of the key factors on the mechanism and variability of the MOK, which should be taken into consideration for the ISM research and understanding of it’s onset variability. Although, it is well known that the ISM is associated with a meridional land–sea thermal contrast reinforced by the thermal effects of the elevated TP. And Zhang (2001) already mentioned that strong (weak) water vapor transport from the Indian monsoon region is accompanied by less (more) water vapor transport over the east Asia, leading to less (more) rainfall over the middle and lower reaches of the Yangtze River valley^24^.

## Data and Methods

Several monthly and daily data sets are employed in this study. Daily interpolated SST data from National Oceanic and Atmospheric Administration (NOAA)^25^ are used in this study. We also used the daily mean interpolated OLR data from NOAA^26^, daily and monthly mean of SLP, wind fields, air temperature, specific humidity and daily mean of Omega from the National Centers for Environmental Prediction (NCEP)-National Center for Atmospheric Research (NCAR) reanalysis data set^27^, the monthly mean Hadley Center SST data set^28^, and European Center for Medium Range Weather Forecasts Interim Reanalysis (ERA-Interim) monthly total cloud cover data^29^.

The MOK dates from 1979 to 2013 are taken from the list of onset dates based on the Western Circulation Index (WCI) by Ordonez *et al*.^30^. The onset dates for the year 2009, 2014, 2015, 2016 and 2017, which are not included in Ordonez *et al*.^31^ are taken from the MOK dates as given by the IMD. The normal date of the ISM onset or MOK is chosen as 1^st^ June based on the IMD. The early onset years (hereafter ‘early onset’) are the years when onset occurs at-least 5 days or more prior to 1^st^ June, and the late onset years (hereafter it will be called as ‘late onset’) are the years when onset delays by at-least 5 days or more^10^. Therefore, during 1979 to 2017, 14 early onset and 9 late onset years are selected, which is given in Table 1. To find the difference and evolution pattern prior to the MOK onset date for early and late onset years, a composite analysis of the key meteorological variables for the month of May and 10 days daily evolution prior to the onset are employed. The significance for all the differences is tested with a two tailed *t*-test.

Here, we calculate the moisture convergence and temperature advection and the moisture budget are estimated by applying mass continuity equation and conservation of water vapor in pressure (*p*) coordinates:

1 $$\frac{\partial q}{\partial t}+\nabla .(q{V}_{h})+\frac{\partial }{\partial p}(q\omega )=E-P$$

where V_h_ = (*u*, *v*); u and v represent the zonal and meridional component of the horizontal wind. Equation (1) represents the moisture budget for an air parcel, where the terms represent the local rate of change of q, horizontal moisture flux divergence, vertical moisture flux divergence, evaporation and precipitation rates (source and sink terms) respectively. The horizontal moisture flux convergence (MFC) which is simply the negative horizontal moisture flux divergence, can be written as:

2 $$MFC=-u\frac{\partial q}{\partial x}-v\frac{\partial q}{\partial y}-q(\frac{\partial u}{\partial x}+\frac{\partial v}{\partial y})$$

In Eq. (2), the first two terms are the *advection term*, which represent the horizontal advection of specific humidity and last two terms denote the *convergence term*, which is the product of specific humidity and horizontal mass convergence. Temperature advection is also employed in this study and a constant pressure surface temperature advection can be written as:

3 $$\frac{\partial T}{\partial t}=-\,(u\frac{\partial T}{\partial x}+v\frac{\partial T}{\partial y})$$

where T is air temperature. All the datasets are from 1979 to 2017 except SST, precipitation and moisture convergence. The daily mean SST datasets are dated from 1981 to 2016, whereas, precipitation and moisture convergence datasets are taken from 1979 to 2015 due to non-availability of data.

## Supplementary information

Supplementary Info
