# Supplementary material for: The role of surface air temperature over the east Asia on the early and late Indian Summer Monsoon Onset over Kerala
Source: Sci Rep. 2019 Aug 13;9:11756. doi: 10.1038/s41598-019-47945-4 (PMC6692365; doi:10.1038/s41598-019-47945-4)
Supplement: Supplementary file 1 — Supplementary Info [file 41598_2019_47945_MOESM1_ESM.pdf]

The role of surface air temperature over East Asia on the early and late Indian Summer Monsoon Onset over Kerala

Devanil Choudhury[1,2], Debashis Nath[1*], Chen Wen[1]

[1] Center for Monsoon System Research, Institute of Atmospheric Physics, Chinese Academy of Sciences, Beijing, China

[2] University of Chinese Academy of Sciences

[1]Corresponding Author

Debashis Nath, Professor (Associate)

Center for Monsoon System Research,

Institute of Atmospheric Physics,

Chinese Academy of Sciences,

Beijing, China

Ph: 18310814995

e-mail: debashis@mail.iap.ac.cn

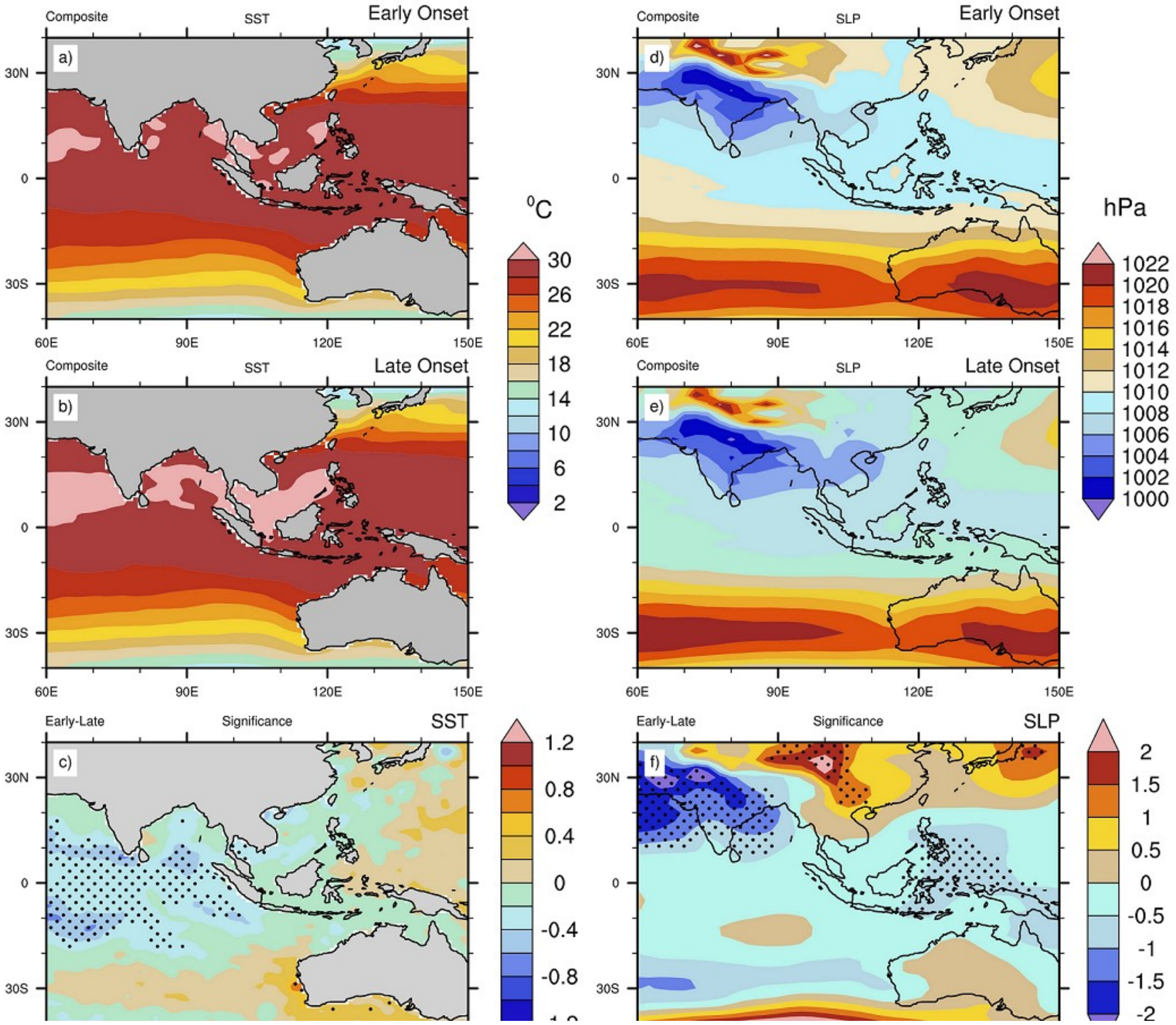

Fig.S1. Composite mean of May SST (^{0}C) and SLP (hPa) for Early, Late onset years and its difference (left and right side respectively). Dots indicate 95% confidence level based on a two tailed *t*-test. The maps in the figure are generated using NCL software [The NCAR Command Language (Version 6.6.2) [Software]. (2019). Boulder, Colorado: UCAR/NCAR/CISL/TDD. http://dx.doi.org/10.5065/D6WD3XH5].

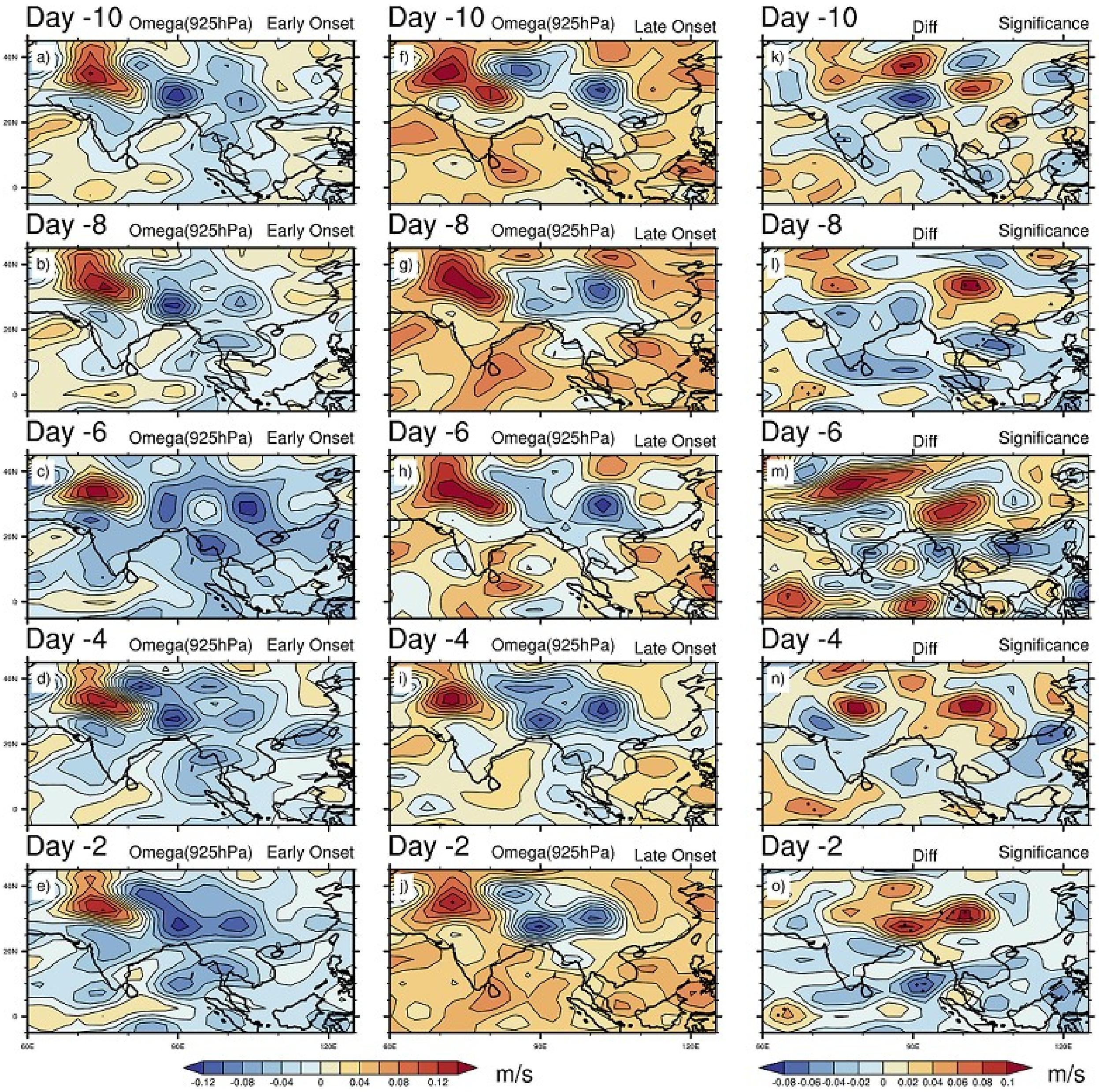

Fig.S2. Composite daily evolution of Omega (m s^{-1}) from 10 days (Day -10) prior to the onset to 2 days before the onset (Day -2) for Early (extreme left), Late onset (middle) and its differences (extreme right). Dots indicate 95% confidence level based on a two tailed *t*-test. The maps in the figure are generated using NCL software [The NCAR Command Language (Version 6.6.2) [Software]. (2019). Boulder, Colorado: UCAR/NCAR/CISL/TDD. http://dx.doi.org/10.5065/D6WD3XH5].

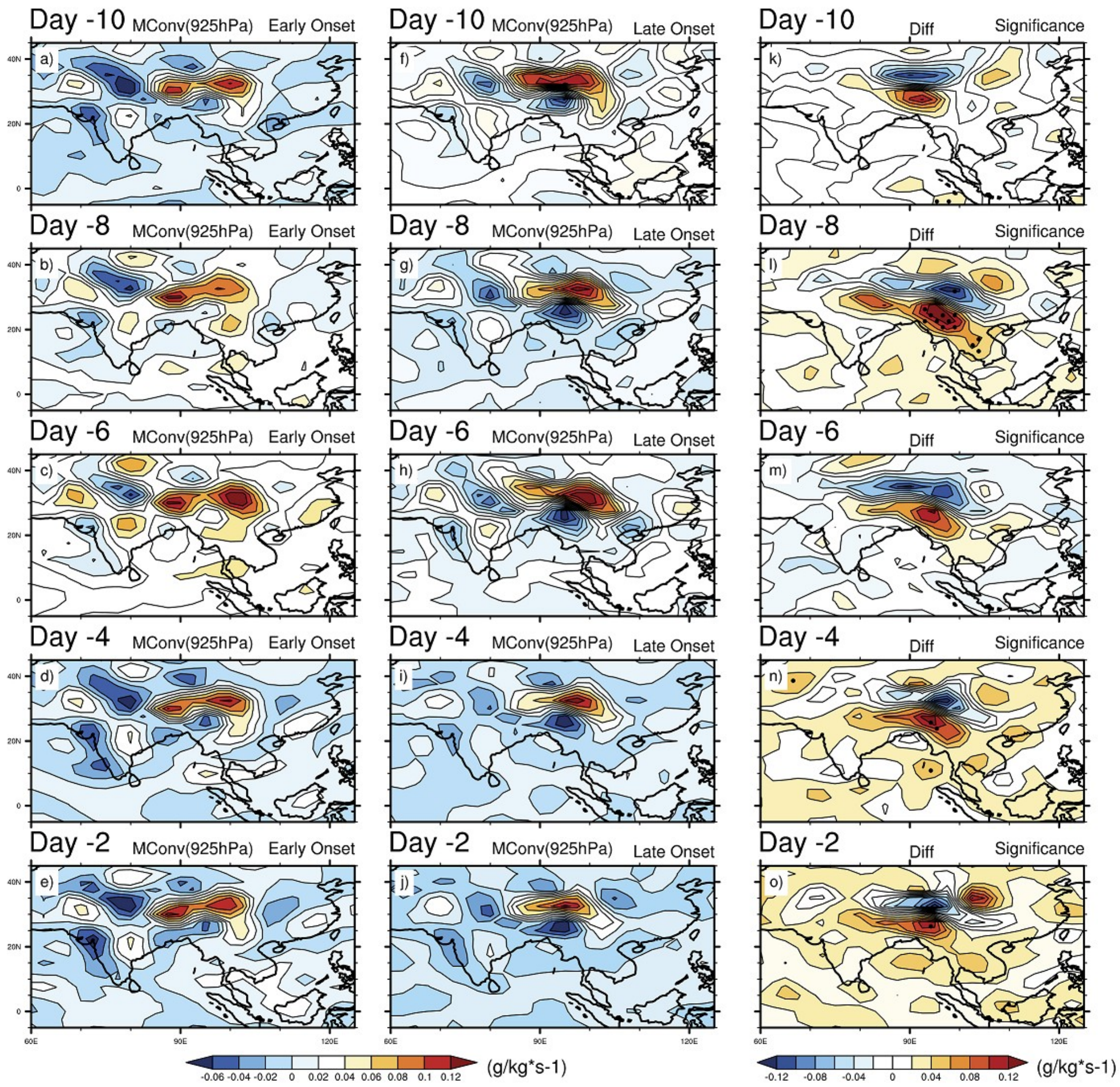

Fig.S3. Composite daily evolution of Moisture Convergence Flux (g/kg*s^{-1}) from 10 days (Day -10) prior to the onset to 2 days before the onset (Day -2) for Early (extreme left), Late onset (middle) and its differences (extreme right). Dots indicate 95% confidence level based on a two tailed *t*-test. The maps in the figure are generated using NCL software [The NCAR Command Language (Version 6.6.2) [Software]. (2019). Boulder, Colorado: UCAR/NCAR/CISL/TDD. http://dx.doi.org/10.5065/D6WD3XH5].

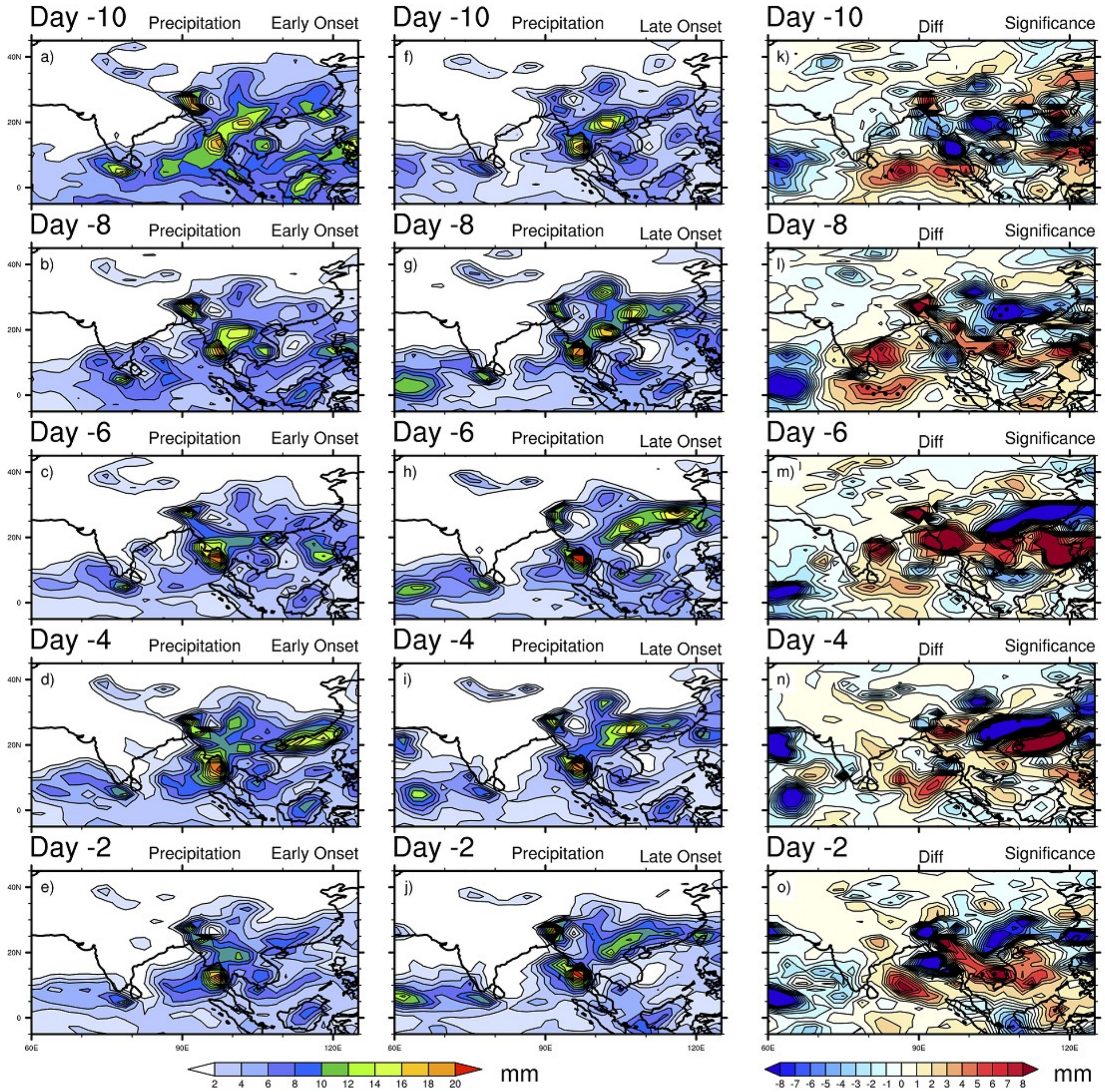

Fig.S4. Composite daily evolution of Precipitation (mm) from 10 days (Day -10) prior to the onset to 2 days before the onset (Day -2) for Early (extreme left), Late onset (middle) and its differences (extreme right). Dots indicate 95% confidence level based on a two tailed *t*-test. The maps in the figure are generated using NCL software [The NCAR Command Language (Version 6.6.2) [Software]. (2019). Boulder, Colorado: UCAR/NCAR/CISL/TDD. http://dx.doi.org/10.5065/D6WD3XH5].

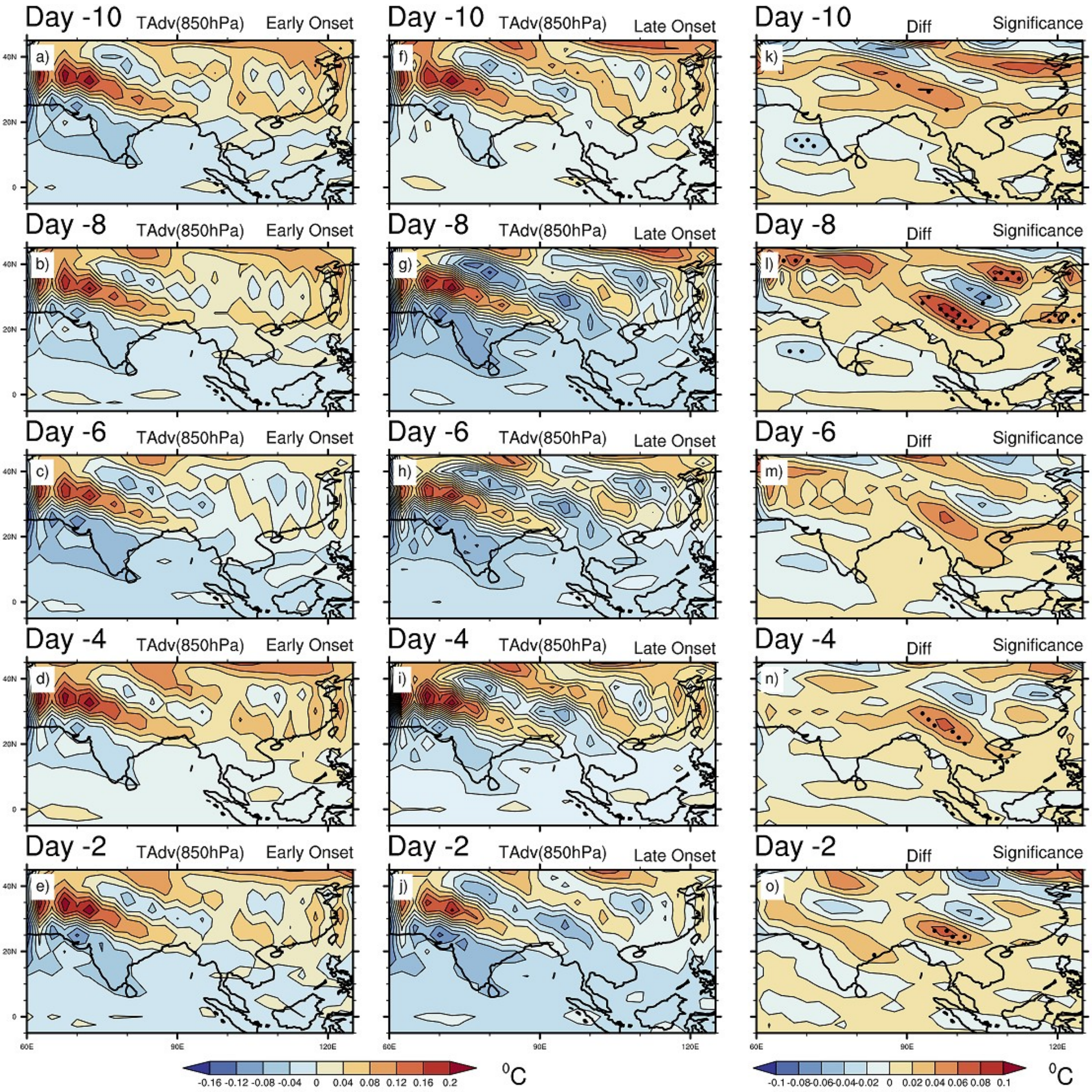

Fig.S5. Composite daily evolution of Horizontal Temperature Advection at 850 hPa (^{0}C) from 10 days (Day -10) prior to the onset to 2 days before the onset (Day -2) for Early (extreme left), Late onset (middle) and its differences (extreme right). Dots indicate 95% confidence level based on two tailed *t*-test. The maps in the figure are generated using NCL software [The NCAR Command Language (Version 6.6.2) [Software]. (2019). Boulder, Colorado: UCAR/NCAR/CISL/TDD. http://dx.doi.org/10.5065/D6WD3XH5].

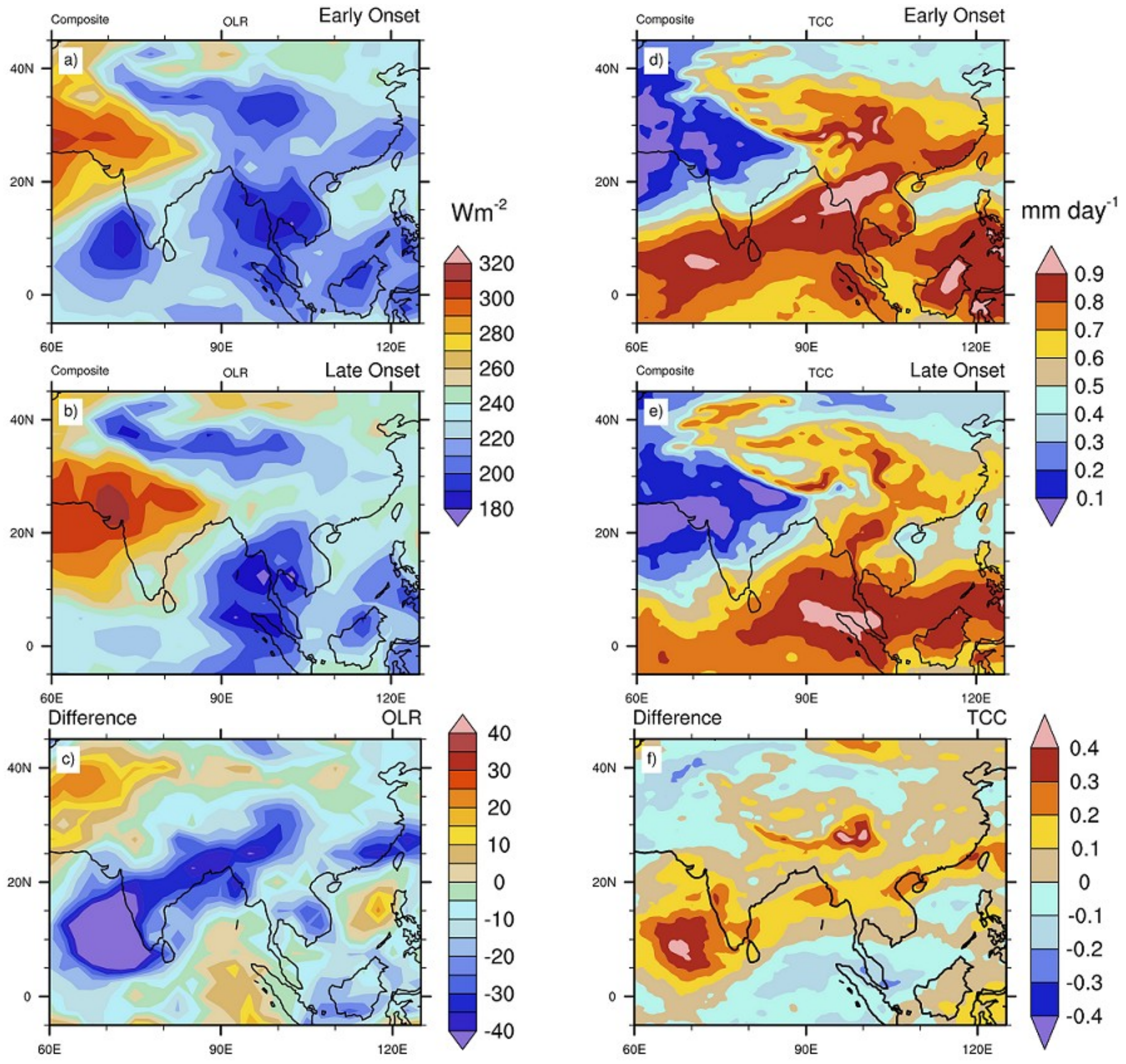

Fig.S6. Composite mean May OLR (Wm^{-2}) and Total Cloud Cover (mm day^{-1}) for an extreme Early (12th May, 1999), Late onset (11th June, 2012) and its difference (left and right side respectively). The maps in the figure are generated using NCL software [The NCAR Command Language (Version 6.6.2) [Software]. (2019). Boulder, Colorado: UCAR/NCAR/CISL/TDD. http://dx.doi.org/10.5065/D6WD3XH5].

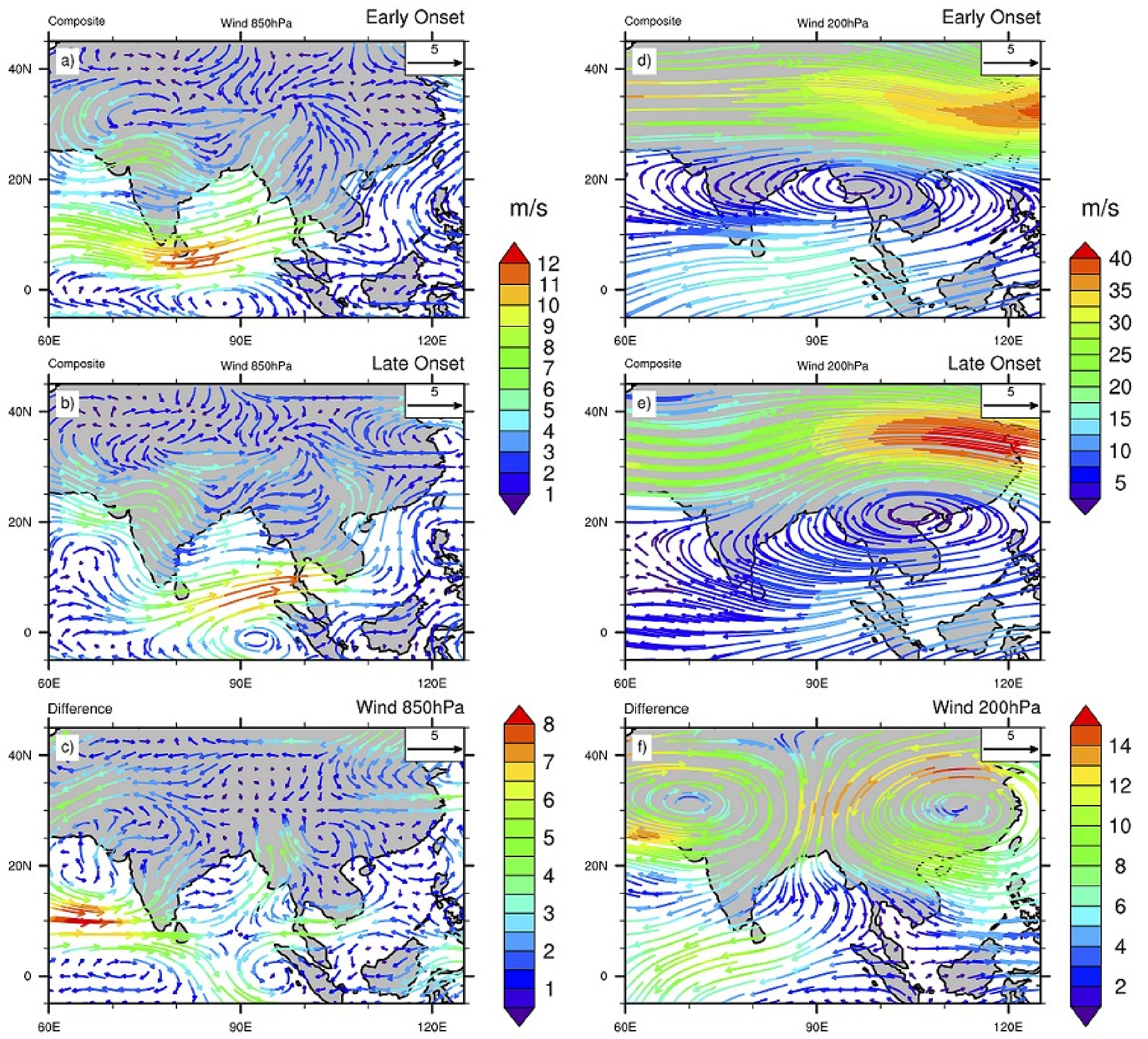

Fig.S7. Composite mean of May wind at 850 and 200 hPa (ms^{-1}) for an extreme Early (12th May, 1999), Late onset (11th June, 2012) and its difference (left and right side respectively). The maps in the figure are generated using NCL software [The NCAR Command Language (Version 6.6.2) [Software]. (2019). Boulder, Colorado: UCAR/NCAR/CISL/TDD. http://dx.doi.org/10.5065/D6WD3XH5].

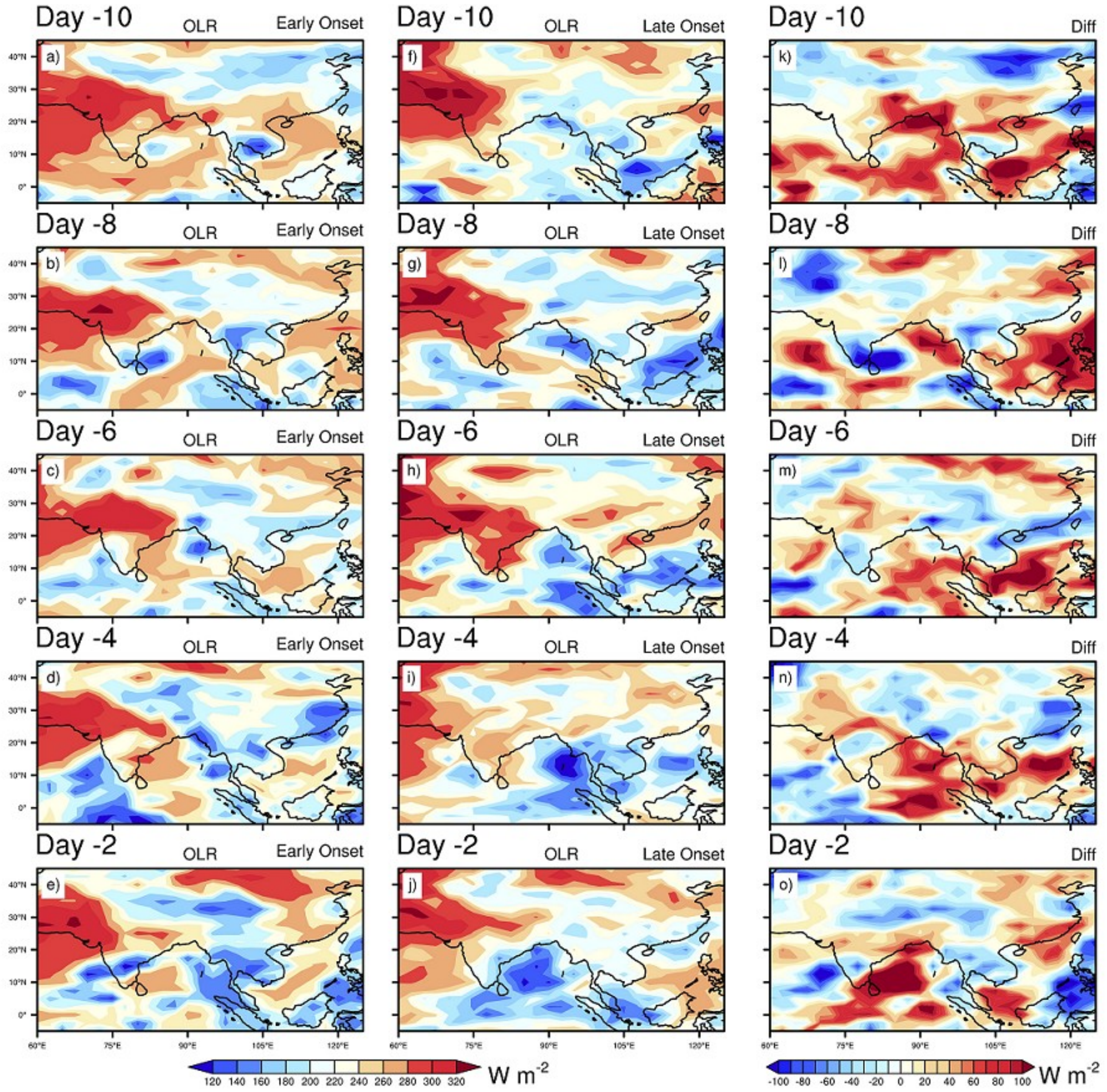

Fig.S8. Composite daily evolution of OLR (Wm^{-2}) from 10 days (Day -10) prior to the onset to 2 days before the onset (Day -2) for an extreme Early (12th May, 1999, extreme left), Late onset (11th June, 2012, middle) and its differences (extreme right). The maps in the figure are generated using NCL software [The NCAR Command Language (Version 6.6.2) [Software]. (2019). Boulder, Colorado: UCAR/NCAR/CISL/TDD. http://dx.doi.org/10.5065/D6WD3XH5].

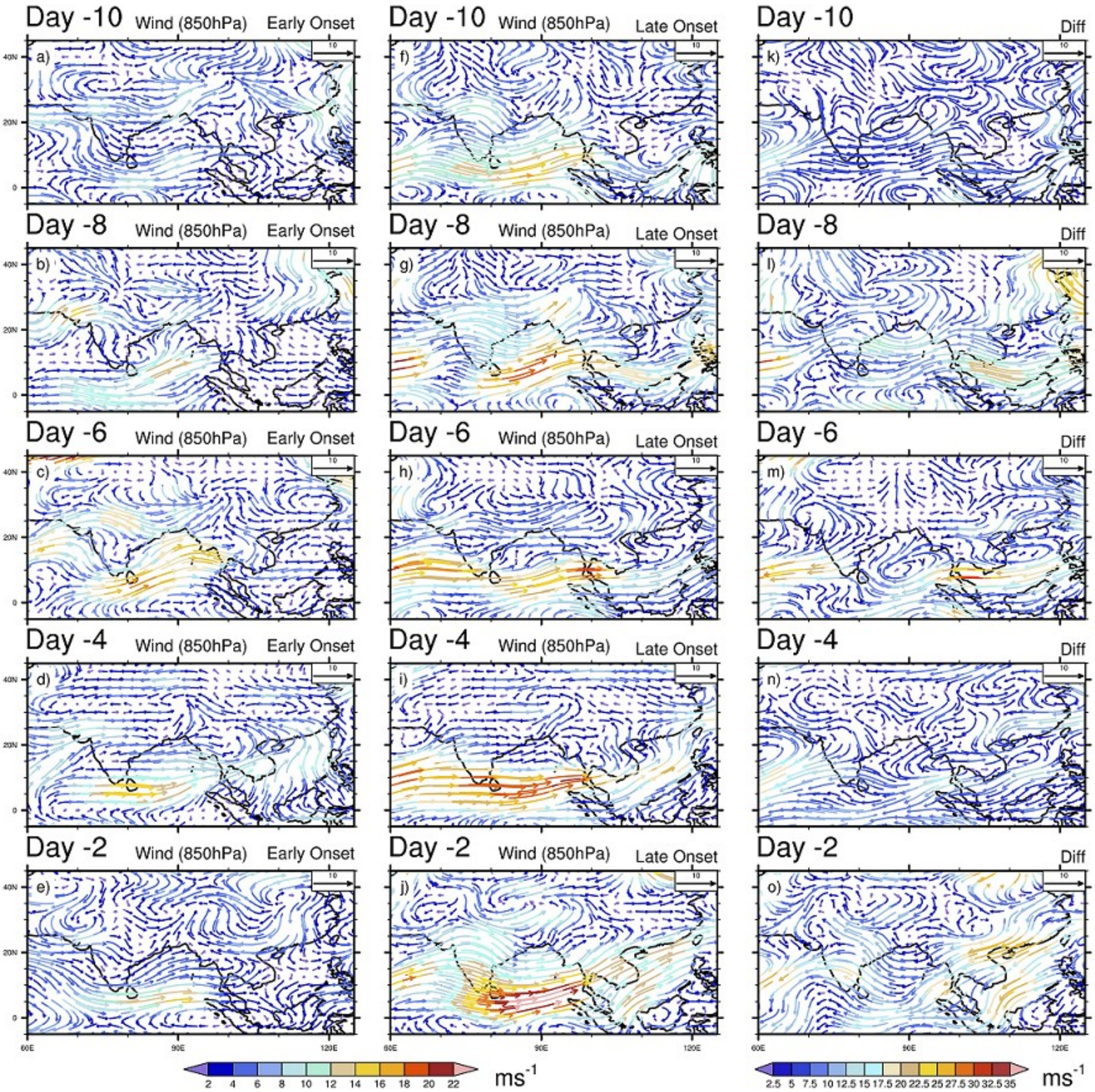

Fig.S9. Composite daily evolution of Wind (ms^{-1}) at 850 hPa from 10 days (Day -10) prior to the onset to 2 days before the onset (Day -2) for an extreme Early (12th May, 1999, extreme left), Late onset (11th June, 2012, middle) and its differences (extreme right). The maps in the figure are generated using NCL software [The NCAR Command Language (Version 6.6.2) [Software]. (2019). Boulder, Colorado: UCAR/NCAR/CISL/TDD. http://dx.doi.org/10.5065/D6WD3XH5].

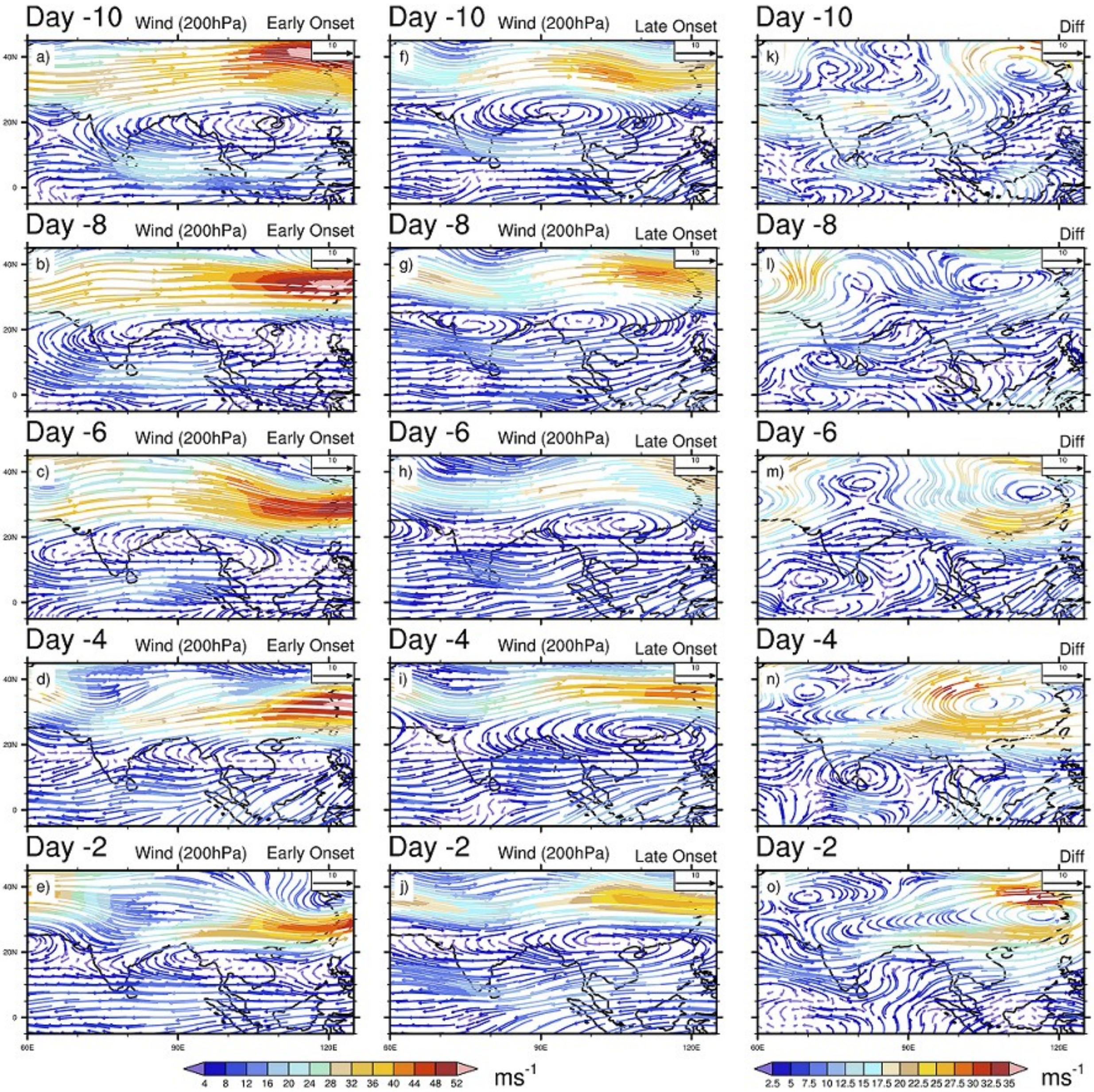

Fig.S10. Composite daily evolution of Wind (ms^{-1}) at 200 hPa from 10 days (Day -10) prior to the onset to 2 days before the onset (Day -2) for an extreme Early (12th May, 1999, extreme left), Late onset (11th June, 2012, middle) and its differences (extreme right). The maps in the figure are generated using NCL software [The NCAR Command Language (Version 6.6.2) [Software]. (2019). Boulder, Colorado: UCAR/NCAR/CISL/TDD. http://dx.doi.org/10.5065/D6WD3XH5].

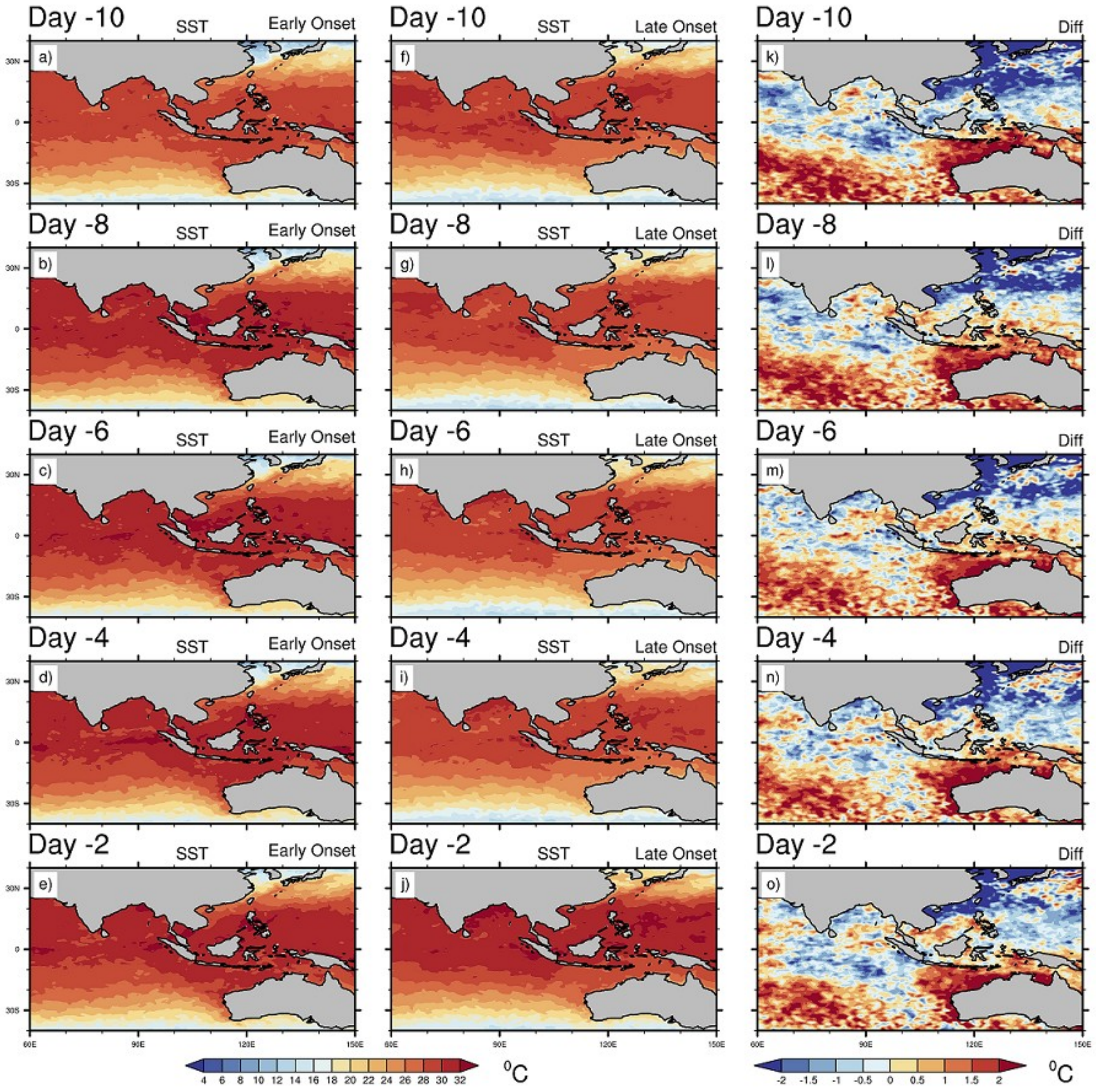

Fig.S11. Composite daily evolution of SST (^{0}C) from 10 days (Day -10) prior to the onset to 2 days before the onset (Day -2) for an extreme Early (12th May, 1999, extreme left), Late onset (11th June, 2012, middle) and its differences (extreme right). The maps in the figure are generated using NCL software [The NCAR Command Language (Version 6.6.2) [Software]. (2019). Boulder, Colorado: UCAR/NCAR/CISL/TDD. http://dx.doi.org/10.5065/D6WD3XH5].

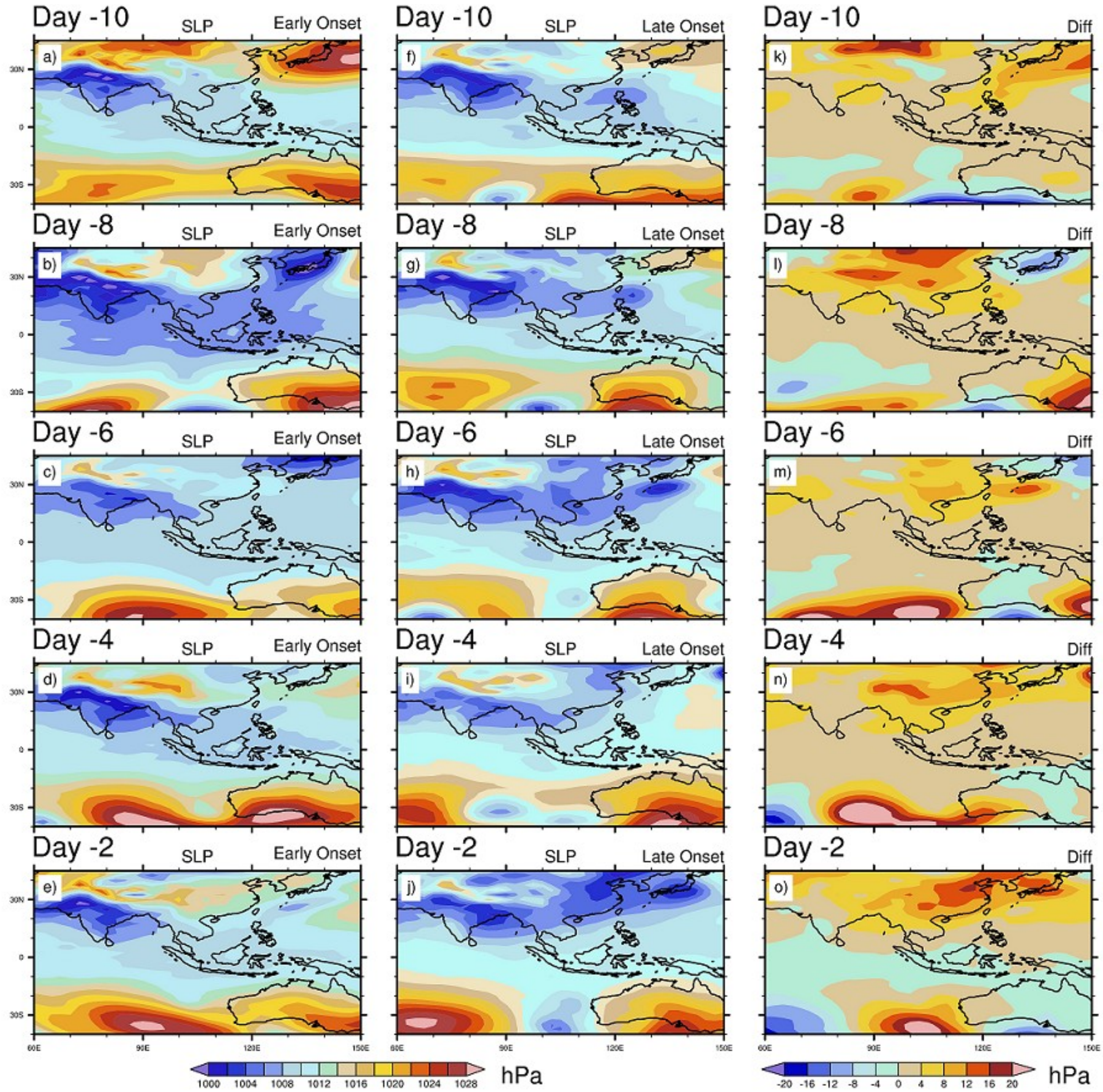

Fig.S12. Composite daily evolution of SLP (hPa) from 10 days (Day -10) prior to the onset to 2 days before the onset (Day -2) for an extreme Early (12th May, 1999, extreme left), Late onset (11th June, 2012, middle) and its differences (extreme right). The maps in the figure are generated using NCL software [The NCAR Command Language (Version 6.6.2) [Software]. (2019). Boulder, Colorado: UCAR/NCAR/CISL/TDD. http://dx.doi.org/10.5065/D6WD3XH5].

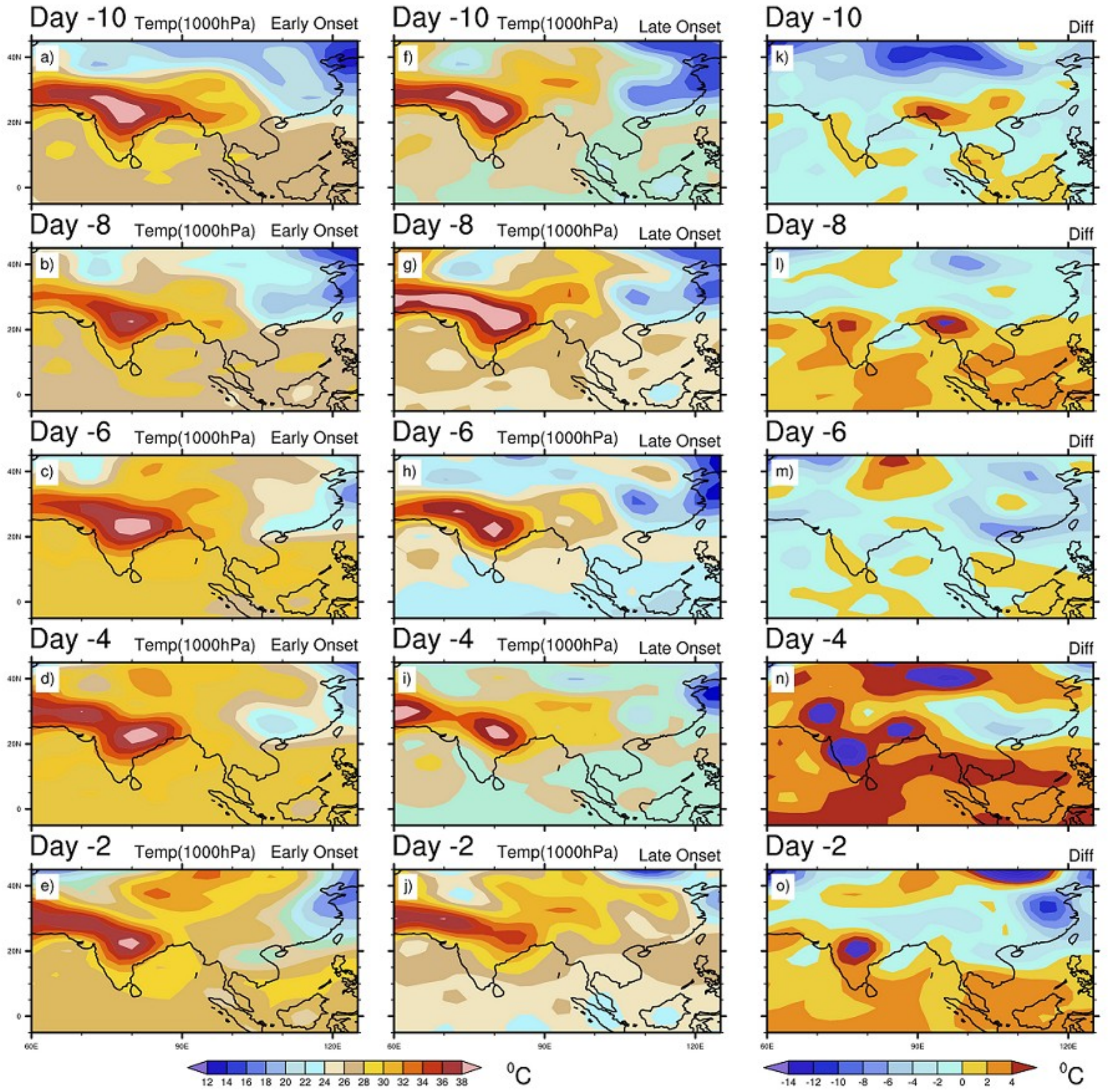

Fig.S13. Composite daily evolution of Surface Air Temperature at 1000 hPa (^{0}C) from 10 days (Day -10) prior to the onset to 2 days before the onset (Day -2) for an extreme Early (12th May, 1999, extreme left), Late onset (11th June, 2012, middle) and its differences (extreme right). The maps in the figure are generated using NCL software [The NCAR Command Language (Version 6.6.2) [Software]. (2019). Boulder, Colorado: UCAR/NCAR/CISL/TDD. http://dx.doi.org/10.5065/D6WD3XH5].

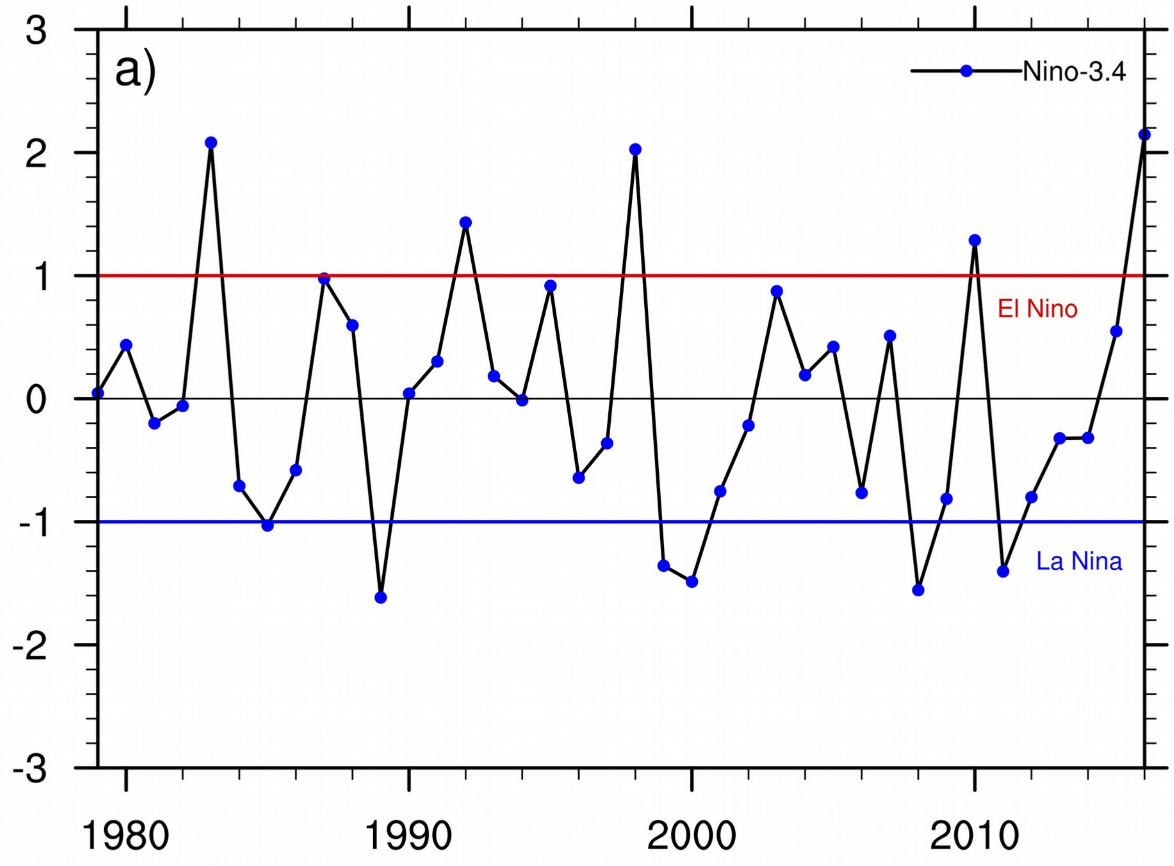

Fig.S14. The inter annual variation of area averaged SST anomalies over eastern Pacific Ocean (Niňo-3.4 region, 5^0S-5^0N, 120^0-170^0W) during winter season (DJF) since 1979 to 2016. The anomalies greater and lesser than 1^0C are considered as El-Niňo and La-Niňa years respectively. The plot in the figure is generated using NCL software [The NCAR Command Language (Version 6.6.2) [Software]. (2019). Boulder, Colorado: UCAR/NCAR/CISL/TDD. http://dx.doi.org/10.5065/D6WD3XH5].

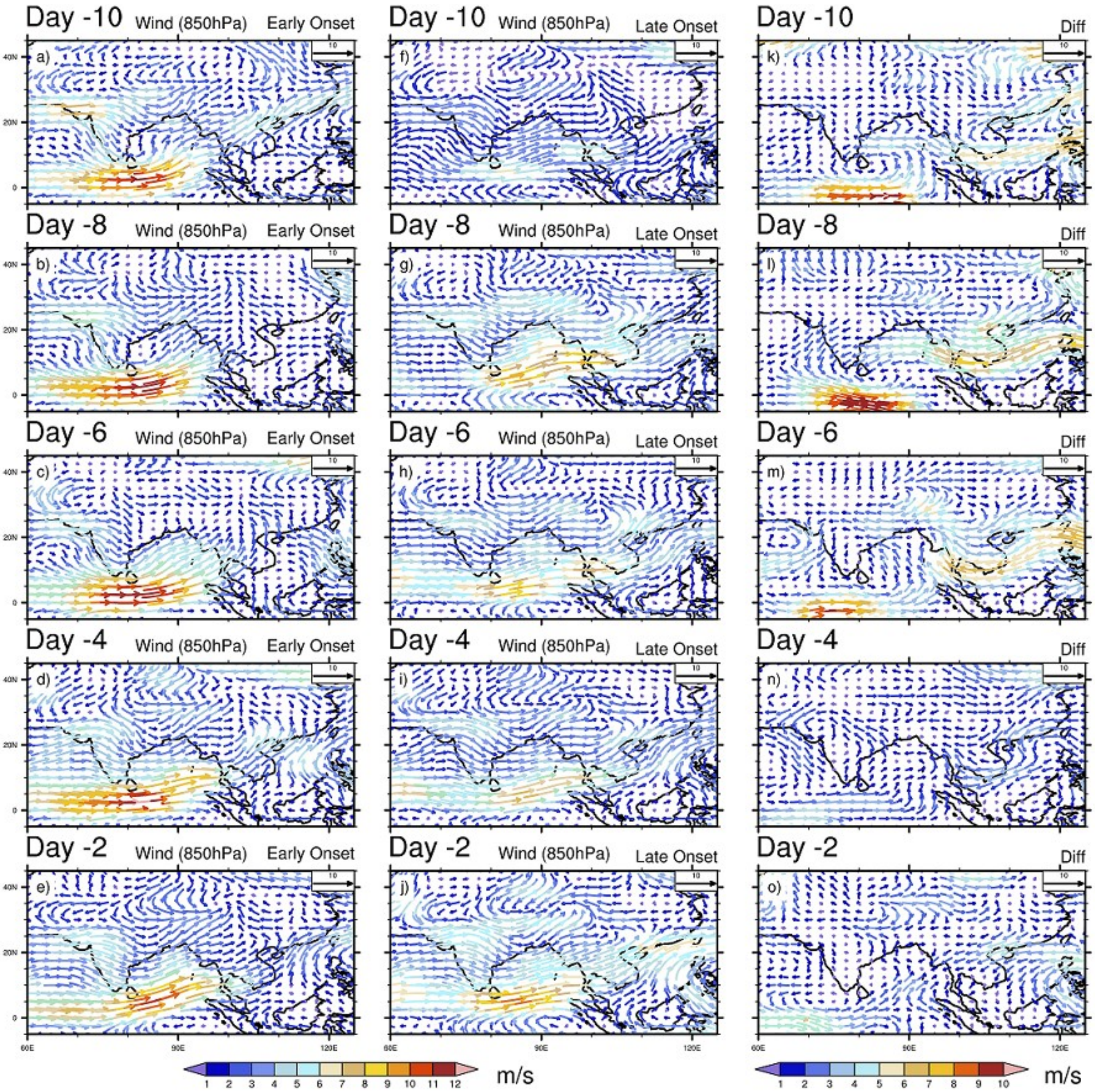

Fig.S15. Composite daily evolution of Wind (m s^{-1}) at 850 hPa from 10 days (Day -10) prior to the onset to 2 days before the onset (Day -2) for Early (extreme left), Late onset (middle) and its differences (extreme right). Here, this new list of onset years are not associated with ENSO years. The maps in the figure are generated using NCL software [The NCAR Command Language (Version 6.6.2) [Software]. (2019). Boulder, Colorado: UCAR/NCAR/CISL/TDD. http://dx.doi.org/10.5065/D6WD3XH5].

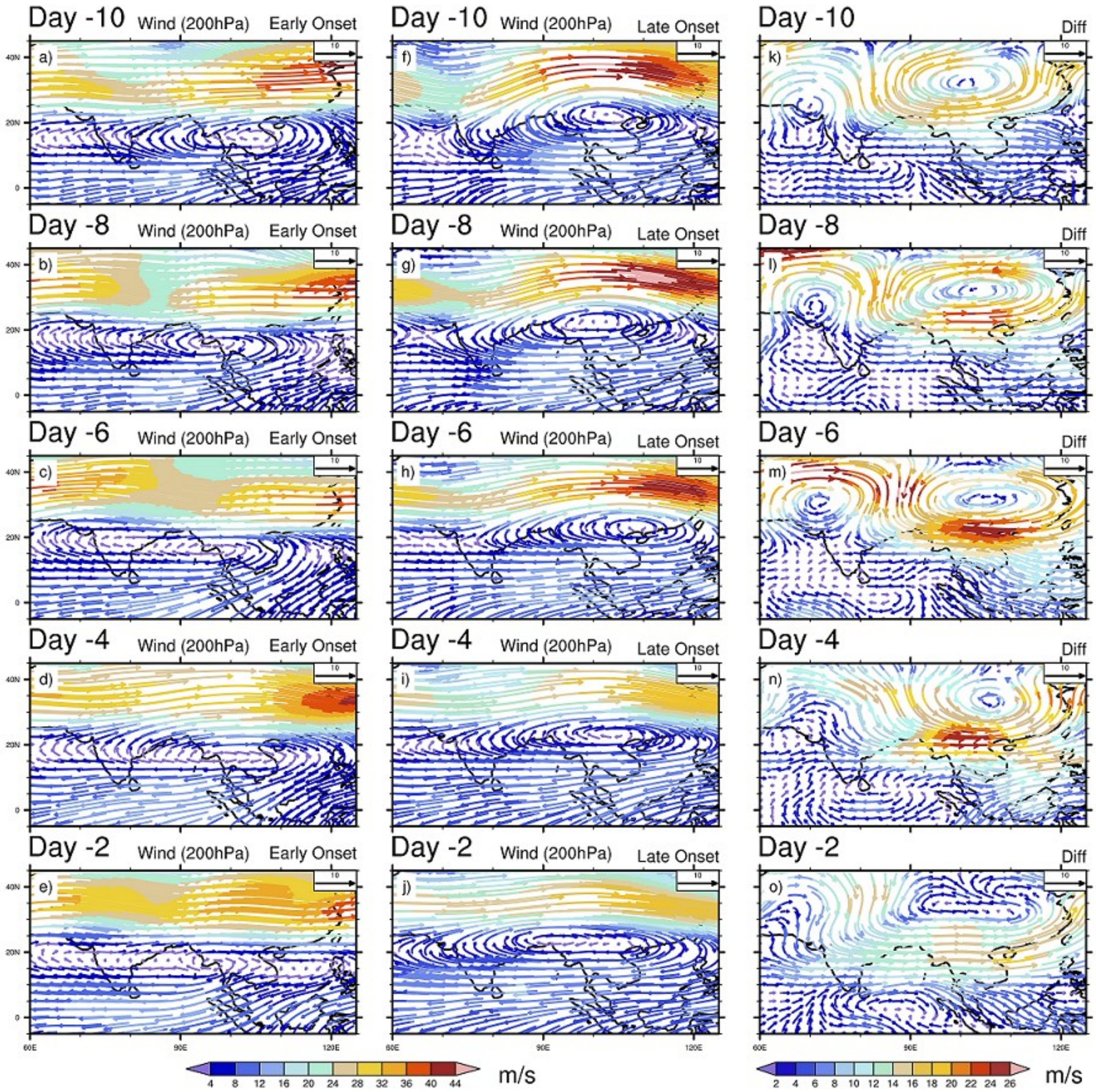

Fig.S16. Composite daily evolution of Wind (m s^{-1}) at 200 hPa from 10 days (Day -10) prior to the onset to 2 days before the onset (Day -2) for Early (extreme left), Late onset (middle) and its differences (extreme right). Here, this new list of onset years are not associated with ENSO years. The maps in the figure are generated using NCL software [The NCAR Command Language (Version 6.6.2) [Software]. (2019). Boulder, Colorado: UCAR/NCAR/CISL/TDD. http://dx.doi.org/10.5065/D6WD3XH5].

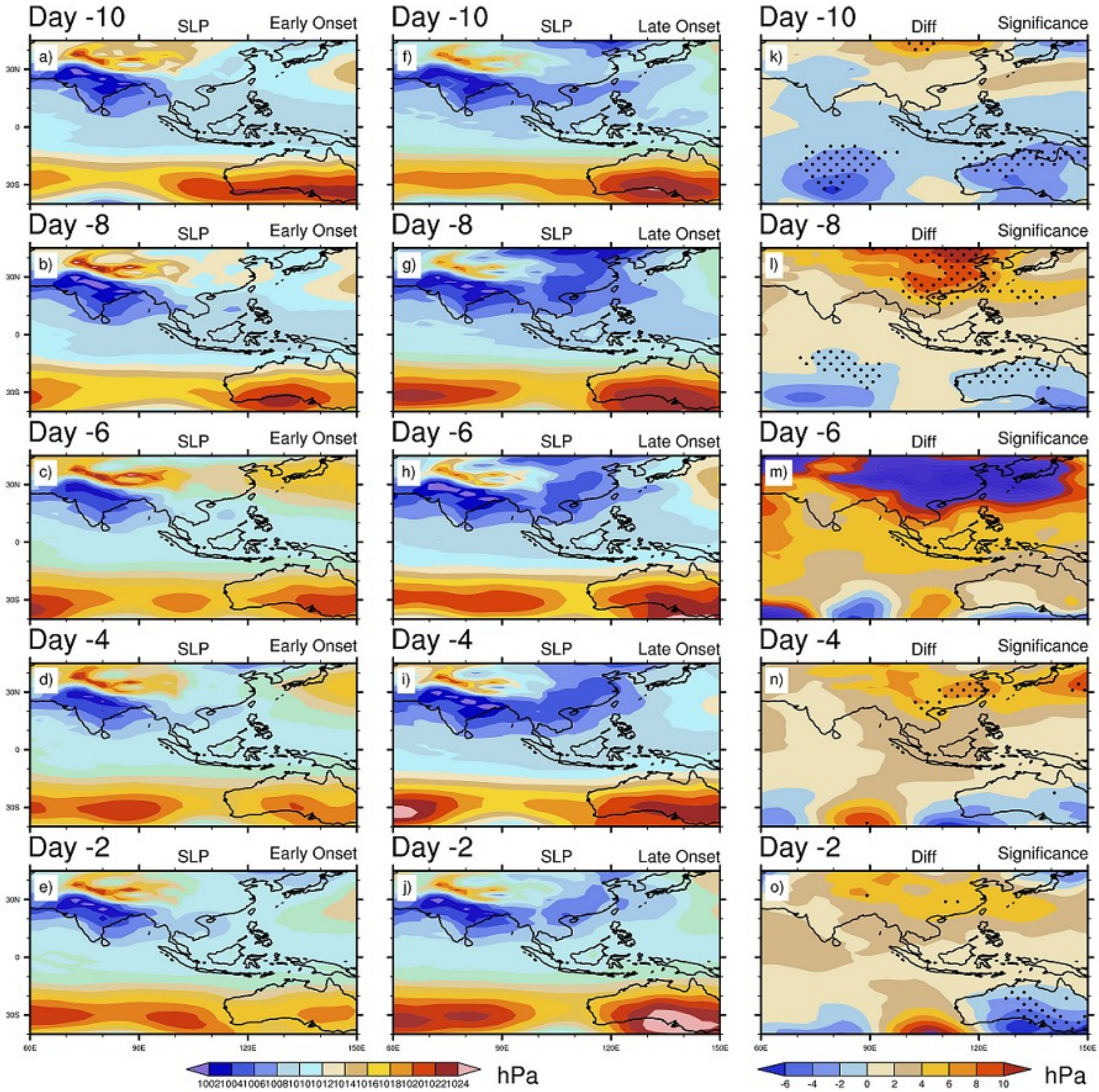

Fig.S17. Composite daily evolution of SLP (hPa) from 10 days (Day -10) prior to the onset to 2 days before the onset (Day -2) for Early (extreme left), Late onset (middle) and its differences (extreme right). Here, this new list of onset years is not associated with ENSO years. Dots indicate 95% confidence level based on a two tailed *t*-test. The maps in the figure are generated using NCL software [The NCAR Command Language (Version 6.6.2) [Software]. (2019). Boulder, Colorado: UCAR/NCAR/CISL/TDD. http://dx.doi.org/10.5065/D6WD3XH5].

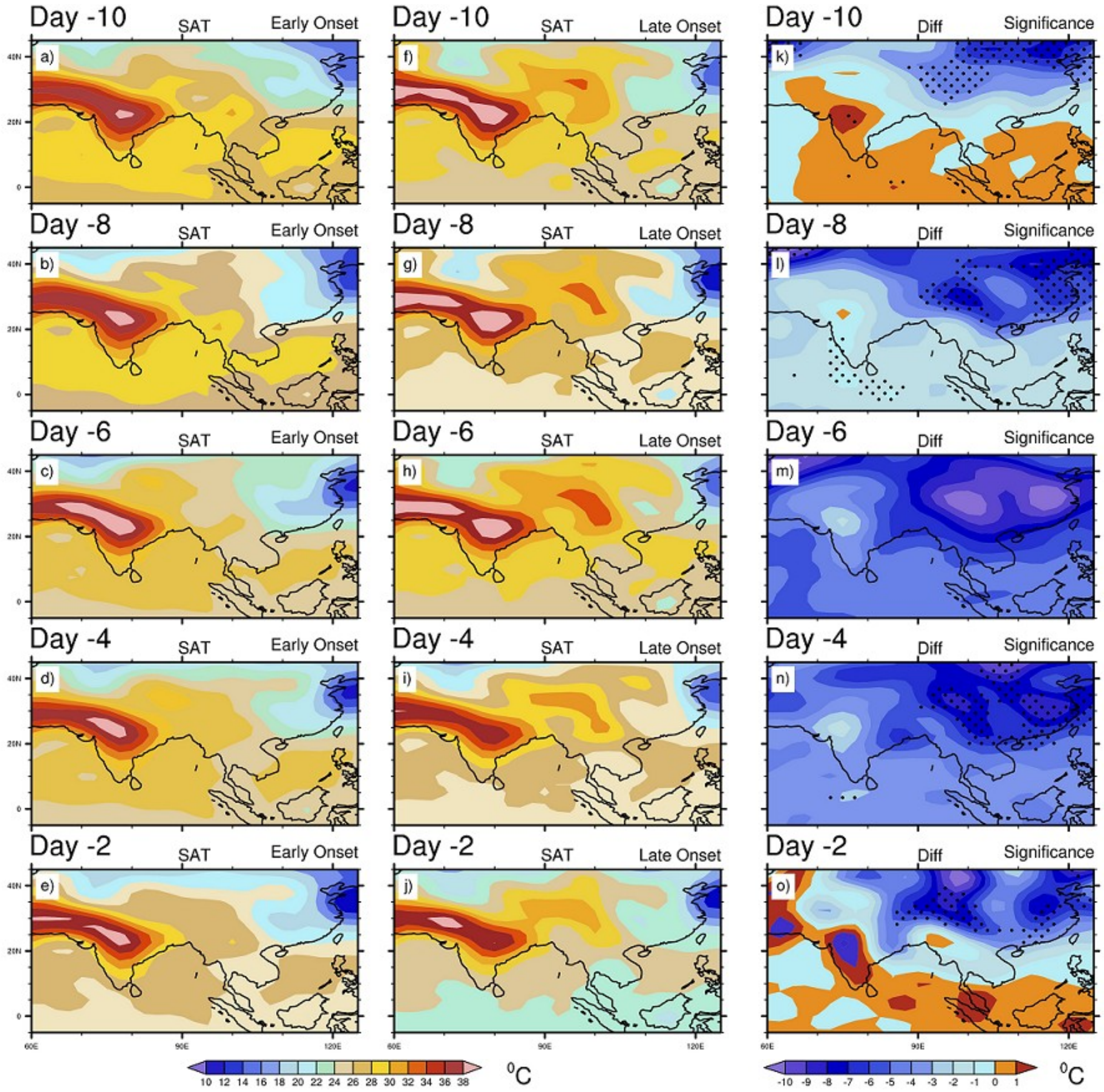

Fig.S18. Composite daily evolution of Surface Air Temperature (^{0}C) from 10 days (Day -10) prior to the onset to 2 days before the onset (Day -2) for Early (extreme left), Late onset (middle) and its differences (extreme right). Here, this new list of onset years are not associated with ENSO years. Dots indicate 95% confidence level based on a two tailed *t*-test. The maps in the figure are generated using NCL software [The NCAR Command Language (Version 6.6.2) [Software]. (2019). Boulder, Colorado: UCAR/NCAR/CISL/TDD. http://dx.doi.org/10.5065/D6WD3XH5].

a)

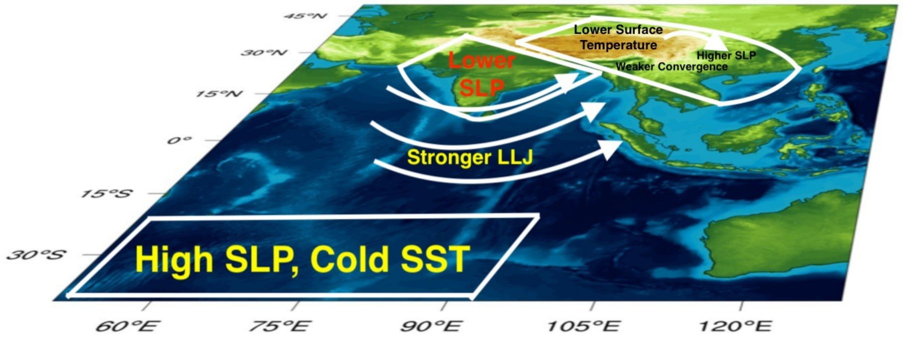

b)

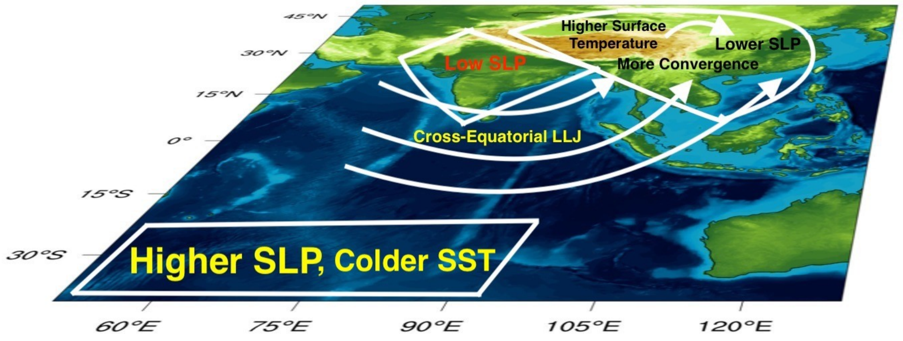

Fig. S19. The schematic plot of typical Early (a) and Late (b) onset. The maps in the figure are generated using NCL software [The NCAR Command Language (Version 6.6.2) [Software]. (2019). Boulder, Colorado: UCAR/NCAR/CISL/TDD. http://dx.doi.org/10.5065/D6WD3XH5].
